# Explainable artificial intelligence (XAI) to find optimal in-silico biomarkers for cardiac drug toxicity evaluation

**DOI:** 10.1038/s41598-024-71169-w

**Published:** 2024-10-14

**Authors:** Muhammad Adnan Pramudito, Yunendah Nur Fuadah, Ali Ikhsanul Qauli, Aroli Marcellinus, Ki Moo Lim

**Affiliations:** 1 Computational Medicine Lab, Department of IT Convergence Engineering, Kumoh National Institute of Technology, Gumi, 39177 Republic of Korea; 2 Computational Medicine Lab, Department of Medical IT Convergence Engineering, Kumoh National Institute of Technology, Gumi, 39177 Republic of Korea; 3 School of Electrical Engineering, Telkom University, Bandung, 40257 Indonesia; 4 Department of Engineering, Faculty of Advanced Technology and Multidiscipline, Universitas Airlangga, Surabaya, 60115 Jawa Timur Indonesia; 5 Meta Heart Co., Ltd., Gumi, 39253 Republic of Korea

**Keywords:** Virtual screening, Machine learning

## Abstract

The Comprehensive In-vitro Proarrhythmia Assay (CiPA) initiative aims to refine the assessment of drug-induced torsades de pointes (TdP) risk, utilizing computational models to predict cardiac drug toxicity. Despite advancements in machine learning applications for this purpose, the specific contribution of in-silico biomarkers to toxicity risk levels has yet to be thoroughly elucidated. This study addresses this gap by implementing explainable artificial intelligence (XAI) to illuminate the impact of individual biomarkers in drug toxicity prediction. We employed the Markov chain Monte Carlo method to generate a detailed dataset for 28 drugs, from which twelve in-silico biomarkers of 12 drugs were computed to train various machine learning models, including Artificial Neural Networks (ANN), Support Vector Machines (SVM), Random Forests (RF), XGBoost, K-Nearest Neighbors (KNN), and Radial Basis Function (RBF) networks. Our study’s innovation is leveraging XAI, mainly through the SHAP (SHapley Additive exPlanations) method, to dissect and quantify the contributions of biomarkers across these models. Furthermore, the model performance was evaluated using the test set from 16 drugs. We found that the ANN model coupled with the eleven most influential in-silico biomarkers namely $\frac{dVm}{dt}_{repol}, \frac{dVm}{dt}_{max}, {APD}_{90}, {APD}_{50}, {APD}_{tri}, {CaD}_{90}, {CaD}_{50}, {Ca}_{tri}, {Ca}_{Diastole}, qInward, and qNet$ showed the highest classification performance among all classifiers with Area Under the Curve (AUC) scores of 0.92 for predicting high-risk, 0.83 for intermediate-risk, and 0.98 for low-risk drugs. We also found that the optimal in silico biomarkers selected based on SHAP analysis may be different for various classification models. However, we also found that the biomarker selection only sometimes improved the performance; therefore, evaluating various classifiers is still essential to obtain the desired classification performance. Our proposed method could provide a systematic way to assess the best classifier with the optimal in-silico biomarkers for predicting the TdP risk of drugs, thereby advancing the field of cardiac safety evaluations.

## Introduction

Torsade de Pointes (TdP) is a hazardous cardiac condition characterized by the twisting of the QRS complex in electrocardiogram (ECG) signals, typically triggered by adverse drug reactions^1,2^. The evaluation of drug-induced cardiac arrhythmias leading to TdP risk has been a pivotal concern for both researchers and the international regulatory board. The Comprehensive In-vitro Proarrhythmia Assay (CiPA) paradigm was introduced to address this issue. CiPA is a novel approach that incorporates in silico simulations and comprehensive evaluation of drug responses across multiple ion channels^3^. CiPA comprises four integral components^4^. The first facet involves in vitro experiments targeting multiple cardiac ionic currents, with a primary focus on hERG, late sodium, and L-type calcium currents, to assess the torsadogenic risk of a drug^4,5^. The second element employs in silico models, utilizing in vitro datasets as input to estimate drug toxicity by mimicking the human ventricular myocyte responses^5^. The third component scrutinizes potential unexpected in vitro effects on human stem cell-derived ventricular cardiac myocytes^5^. Lastly, the fourth facet determines the potential human impact of drugs, considering human-specific metabolic characteristics by examining in vivo ECG biomarkers in phase I clinical trials^5^.

According to FDA requirements, each new drug requires preclinical testing to evaluate its pharmacological activity and acute toxicity in animals^6^. In emergencies involving exposure to hazardous substances, the FDA grants an exemption for the use of the drug in humans based on the results of animal testing under The Animal Efficacy Rule^7^. However, animal testing involves high research costs, and there is a risk of missing drugs that could benefit humans^8^. It happens because the results of preclinical toxicological testing in animals cannot always consistently predict their effectiveness in humans, so the probability of test failure reaches almost 50%^8^. Therefore, in-silico modeling methods have been developed for drug testing that are more consistent, efficient, and can improve human safety. These methods are related to CiPA guidelines, which use computer simulations that mimic the human ventricle to predict the risk of drug toxicity.

The baseline of cardiac cell model that is commonly used for developing CiPA-based TdP risk classifier is originated from the model proposed by O’Hara Rudy et al. (ORd model)^9^. The O’Hara et al. developed a human ventricular action potential model, leveraging new, healthy human ventricular data to include features such as Ca^2+^ and voltage-dependent inactivation of the L-type Ca^2+^ current (ICaL), kinetics for various ion currents like the transient outward, rapid delayed rectifier (IKr), Na^+^/Ca^2+^ exchange (INaCa), and inward rectifier currents. Their model incorporated action potential recordings across physiological cycle lengths and examined the rate dependence and restitution of action potential duration (APD) with and without specific channel blockers. Then, Li et al. introduced a drug-IKr dynamic interaction utilizing the ORd cardiac cell model^10^. Furthermore, Dutta et al. engineered an in silico model employing the CiPAORdv1.0 ventricular myocyte model^9,11^. Dutta et al.’s model fine-tuned the maximal conductance constants of ion channels of the ORd-dynamic-IKr model proposed by Li et al.^10^, explicitly targeting IKs, ICaL, IKr, INaL, and IK1.

Multiple studies have developed drug testing systems based on CiPA guidelines for categorizing the TdP risk associated with drugs. Polak et al. suggested using feature extraction from in-silico biomarkers, such as ${APD}_{90}$, ${APD}_{50}$, Pseudo ECG, QRS width, QT interval, initial repolarization time, and late repolarization time as input to classify drug risk levels. They used an empirical decision tree with the best accuracy of 89%^12^. Liu et al. used the XGBoost algorithm to classify compounds that exhibit hERG inhibitory activity. They obtained an accuracy of 84.4%, and the area under the receiver operating characteristic curve (AUC) was 0.876^13^.

Furthermore, Lancaster and Sobie achieved significant results using a support vector machine (SVM) that takes both AP and $C{a}^{2+}$ inputs to classify drugs as arrhythmogenic or non-arrhythmogenic^14^. This system performed excellently in their research, with an AUC score reaching 0.963 and only a 12.8% misclassification rate. In addition, Yoo et al. proposed classifying drug risk levels by using nine in-silico biomarkers ($\frac{dVm}{dt}_{max}, {AP}_{resting}, {APD}_{90}, {APD}_{50}, {Ca}_{resting}, {CaD}_{90}, {CaD}_{50}, qInward, and qNet$) as input for artificial neural network (ANN) with an AUC result of 0.92 for the high-risk group, 0.83 for the intermediate-risk group, and 0.98 for the low-risk group^15^. Based on the results of previous studies, ANN provides the best performance for classifying drug toxicity risk levels.

In short, using machine learning algorithms, previous research has employed various in-silico biomarkers with solid correlations to TdP risk as input features in predicting drugs’ proarrhythmia risk. Some previous studies have also utilized optimized hERG models in in-silico simulations to determine drug risk levels for TdP. However, despite various machine learning algorithms, including logistic regression, random forest (RF), the Extreme Gradient Boosting (XGBoost), and ANN, being used to classify cardiac toxicity of drugs^12,13, 15^, the primary challenge remains in the analysis of which biomarkers are most crucial in classifying the drugs’ TdP risk level.

In related fields, researchers have also significantly studied optimization and prediction models. For example, a study explores co-design strategies to optimize both structural and dynamic performance parameters simultaneously, resulting in reduced operating costs and improved performance^16^. Similarly, other research demonstrates the application of bio-inspired optimization algorithms in parameter identification, achieving high accuracy and robustness in the identified parameters^17^. Another innovative approach is the bio-inspired optimization algorithm applied to various optimization problems, showing significant improvements in solution quality and computational efficiency^18^.

While these examples use sophisticated optimization algorithms tailored to specific engineering problems, in this research, we employ grid search (GS) to optimize hyperparameters for machine learning models to predict TdP risk. GS is an exhaustive search method that tunes hyperparameters to find the optimal model configuration by evaluating all possible combinations within a specified range. This method ensures that the selected model parameters lead to the best possible predictive performance.

Several methods have been developed to explain the predictive behavior of machine learning models and one of the most prominent is the SHapley Additive explanations (SHAP) method. The SHAP method was introduced in 2017 by Lundberg et al.^19^ to interpret the complex machine learning models by unifying the six existing methods namely LIME, DeepLIFT, Layer-Wise Relevance Propagation, and three classic Shapley value estimation methods (Shapley regression values, Shapley sampling values, and Quantitative Input Influence)^20–26^. Some example uses of the SHAP method were demonstrated by Lundberg et al.^27^ in making a tree explanation method known as the Tree Explainer to solve some medical machine learning problems. The authors demonstrated that the proposed method could allow researchers to identify important but rare mortality risk factors in the general population of the US, find different sub-groups sharing similar risk factors, highlight interaction effects among risk factors for chronic disease of the kidney, and examine features that downgrade the machine learning model’s performance over time. Therefore, given the capability of the SHAP method to interpret the predictive mechanism of the machine learning model, we propose a systematic method to incorporate the SHAP method for evaluating the level of importance of in silico biomarkers for predicting TdP risk of drugs, as well as assessing and improving the predictive performance of the classifiers.

## Methods

**Fig. 1:**
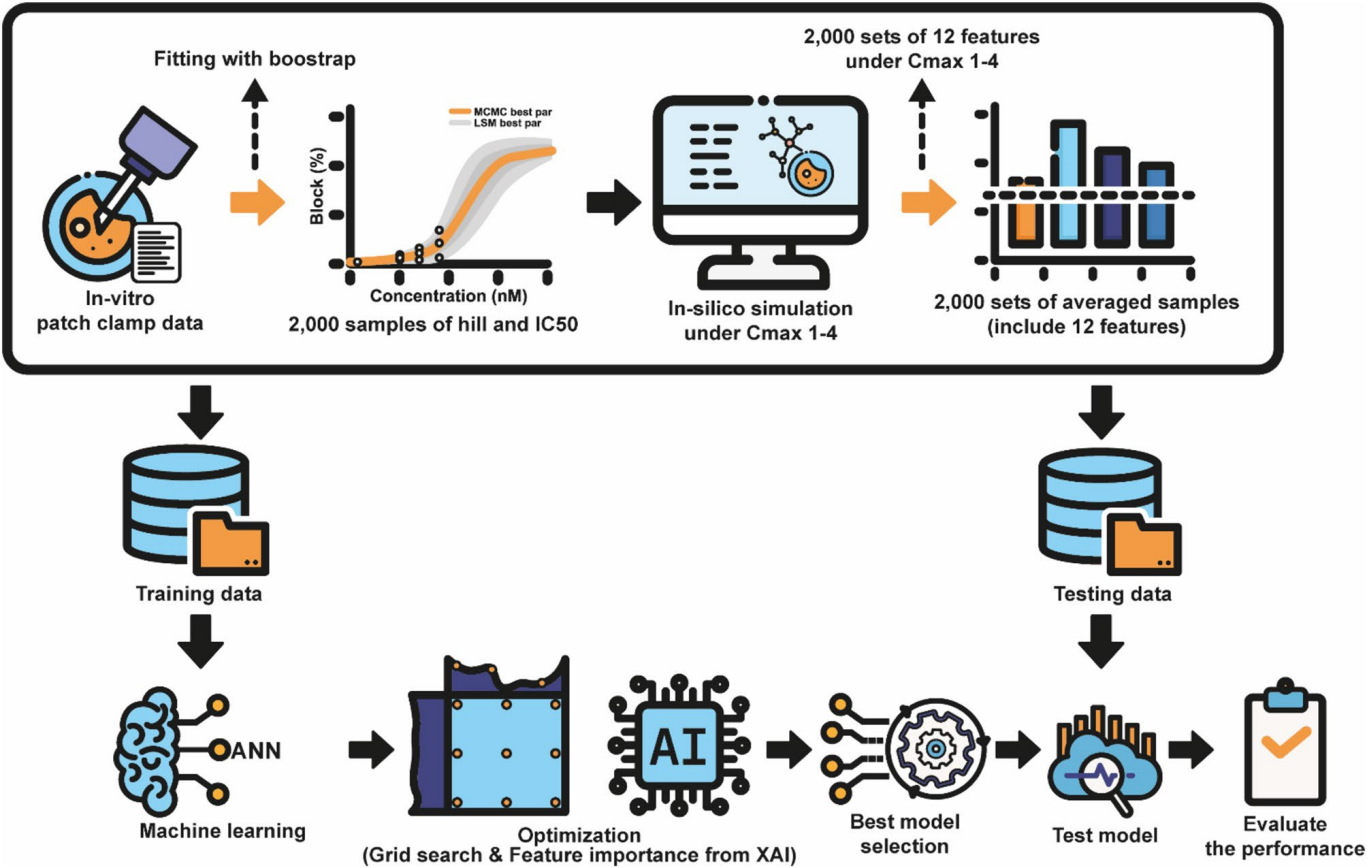
Illustration of our proposed algorithm for evaluating proarrhythmic drug risk, identifying key biomarkers in high, intermediate, and low-risk groups. The figure outlines the process, including preprocessing in-vitro data, sample generation, feature variability simulation, and identifying significant biomarkers using XAI algorithms (ANN). System performance is evaluated through model testing based on the XAI approach.

In this study, we present an algorithm to evaluate proarrhythmic drug risk by identifying the most significant biomarkers contributing to high, intermediate, and low-risk groups. Figure 1 illustrates the entire process of our proposed method. The block diagram consists of several steps, including preprocessing in-vitro experimental data to generate 2000 samples for each drug and using in-silico simulation to generate variability of twelve in-silico biomarkers. Finally, the system performance is evaluated by testing several machine learning models based on the explainable artificial intelligence (XAI) approach.

### In-silico simulation

This study employed in-vitro patch clamp experiments for 28 drugs (see Table 1) sourced from the CiPA group’s dataset (https://github.com/FDA/CiPA/tree/Model-Validation-2018/Hill_Fitting/data). The dataset comprises dose–response inhibition effects of drugs on various ion channels, including calcium channels (${I}_{CaL}$), hERG channels (${I}_{Kr}$), inward rectifying potassium channels (${I}_{K1}$), delayed slow rectifying potassium channels (${I}_{Ks}$), Kv4.3 channels (${I}_{to}$), late sodium channels (${I}_{NaL}$), and peak sodium channels (${I}_{Na}$)^3^. For each drug and ion channel, we collected multiple measurements to capture the variability in response. The 28 in vitro patch clamp experiments provided by CiPA consisted of recordings capturing the dose–response relationships of each drug on the specified ion channels. The input format included information on the drug, ion channel, concentration levels, and corresponding ion channel responses. This detailed dataset served as the foundation for our subsequent analyses.

**Table 1:** List of 28 drugs along with their corresponding C_max_ values.

Proarrhythmic risk level	Train drug		Test drug	
	Drug name	C_max_ (nM)	Drug name	C_max_ (nM)
High risk	Sotalol	14,690	Disopyramide	742
	Dofetilide	2	Ibutilide	100
	Bepridil	33	Vandetanib	255
	Quinidine	3237	Azimilide	70
Intermediate	Cisapride	2.6	Clarithromycin	1206
	Terfenadine	4	Clozapine	71
	Chlorpromazine	38	Domperidone	19
	Ondansetron	139	Droperidol	6.3
			Pimozide	0.43
			Astemizole	0.26
			Risperidone	1.81
Low	Verapamil	81	Metoprolol	1800
	Ranolazine	1948.20	Nifedipine	7.7
	Diltiazem	122	Nitrendipine	3.02
	Mexiletine	4129	Tamoxifen	21
			Loratadine	0.45

Determining the number of 2000 hill curves estimated via Markov-Chain Monte Carlo (MCMC) was a crucial aspect of our study. We aimed for a sufficient sample size to ensure the robustness and reliability of our analyses. The decision to use 2000 samples was based on careful consideration of statistical power, computational feasibility, and the need to adequately represent the underlying probability distribution of Hill equation parameters (IC50 and Hill coefficient) for each drug and ion channel combination^28^. This approach allowed us to evaluate comprehensively how each drug interacts with specific ion channels.

MCMC simulations were applied to generate 2000 samples representing the combined probability distribution of IC50 and Hill coefficient^28^. These samples collectively formed the combined probability distribution used for subsequent analyses. Furthermore, the dose–response inhibition effect of a drug can be expressed mathematically as follows:

1 $$inhibition factor= \frac{1}{1+{\left(\frac{{IC}_{50}}{\left[D\right]}\right)}^{h}}$$

where, ${IC}_{50}$ is the concentration at which 50% inhibition of ion currents occurs, $D$ represents the drug concentration, and $h$ represents the Hill coefficient^9,29^. The inhibition effect of a drug on an ion channel is assumed to rescale the maximum conductance of the ion channel as follows:

2 $${g}_{i}={g}_{i,control}\left(1-inhibition factor\right)$$

where the ${g}_{i}$ is the maximum conductance of ion channel $i$ under drug effects, whereas the ${g}_{i,control}$ is the maximum conductance of ion channel $i$ without drug effect. This comprehensive approach allowed us to evaluate how each drug interacts with specific ion channels. Subsequently, drug effects were simulated at four different concentration levels, ranging from 1 to 4 times the maximal plasma concentration (C_max_) for each drug. The C_max_ values used in our simulations were obtained from relevant literature and experimental data specific to each drug, ensuring accuracy in our concentration-level simulations. Before drug administration, the cell model was simulated without drug effects for 1000 stimulations with a cycle length of 2000 ms until reaching a steady state. Following steady-state achievement, the drug effect was administered with the same number of stimulations and cycle length.

**Fig. 2:**
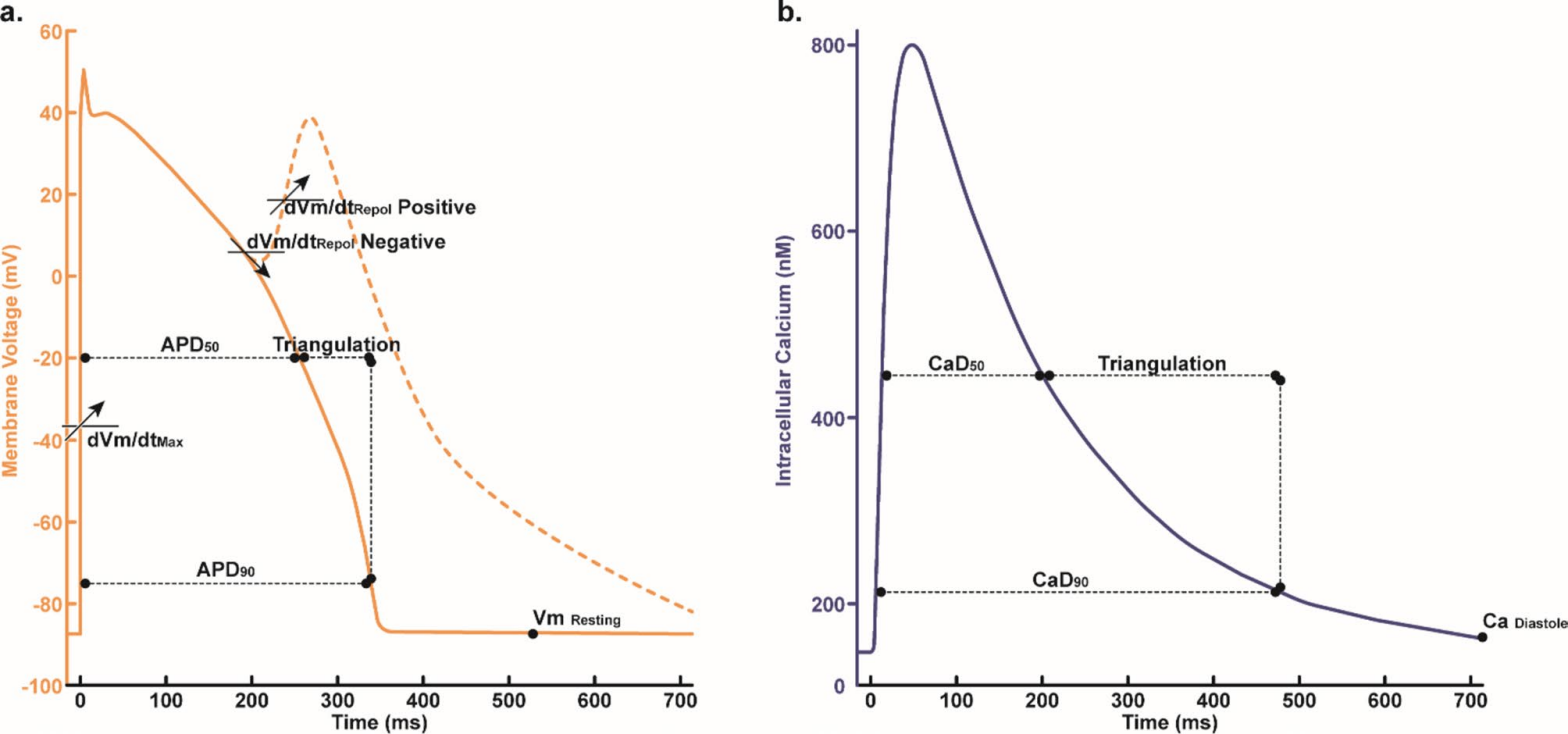
Illustration of in-silico biomarkers in AP profile and Ca profile, which consisted of repolarization $\frac{dVm}{dt}_{repol}, \frac{dVm}{dt}_{max}, { Vm}_{resting}, {APD}_{90}, {APD}_{50}, {APD}_{tri}, {CaD}_{90}, {CaD}_{50}, {Ca}_{tri}, {Ca}_{Diastole}, qInward,$ and $qNet$.

The action potential (AP) selection procedure was employed to obtain AP and Calcium Profile (CP), as shown in Fig. 2, for each drug sample. Following the AP selection mechanism proposed by Chang et al.^28^, the AP with the highest maximum repolarization rate among the last 250 stimulations was selected. The maximum depolarization rate for AP that fully repolarizes can be found between 30 and 90% repolarization. For AP that can repolarize 30% but not 90%, the maximum depolarization rate can be obtained between 30% repolarization until the end of the beat. The maximum repolarization rate for AP that cannot achieve 30% repolarization can be found between the AP peak and the beat’s end. The APs that fail to depolarize were omitted from the analysis. Finally, the APs resulting from the AP selection procedure were used to extract the in-silico biomarkers.

### Biomarker extraction

In our study, we selected twelve in-silico biomarkers, namely $\frac{dVm}{dt}_{repol}$ (repolarization slope), $\frac{dVm}{dt}_{max}$ (maximum depolarization slope), ${Vm}_{resting}$ (resting membrane potential), ${APD}_{90}$ (action potential duration at 90% repolarization), ${APD}_{50}$ (duration at 50% repolarization), ${APD}_{tri}$ (triangle area between $AP{D}_{90}$ and $AP{D}_{50}$), ${CaD}_{90}$ (calcium transient duration at 90% repolarization), ${CaD}_{50}$ (duration at 50% repolarization), ${Ca}_{tri}$ (triangle area between CaD_90 and CaD_50), ${Ca}_{Diastole}$ (diastolic calcium concentration), qInward (Eq. 3), and qNet (Eq. 4). These were generated through in-silico simulations using 2000 sets of ${IC}_{50}$ and Hill coefficients from MCMC analysis. Building on the work of Yoo et al., nine of these biomarkers have been proven significant for predictive performance with ANN Yoo et al.^15^. We incorporated three additional biomarkers: $AP{D}_{tri}$, $\frac{dVm}{dt}_{repol}$, and $C{a}_{tri}$, informed by the findings of Jeong, Danadibrata et al., and Parikh et al., to enhance machine learning performance^30,31,32^.

3 $$qInward= \left(\frac{{I}_{{CaL}_{AUC under Drug}}}{{I}_{{CaL}_{AUC control Drug}}}+\frac{{I}_{{NaL}_{AUC under Drug}}}{{I}_{{NaL}_{AUC control Drug}}}\right)$$

4 $$qNet= {\int }_{0}^{BCL}\left({I}_{NaL}+{I}_{CaL}+{I}_{Kr}+{I}_{Ks}+{I}_{K1}+{I}_{to}\right)dt$$

The selection was driven by their relevance to drug effects, electrophysiological characteristics, and calcium handling in cardiac cells. For instance, the repolarization slope ($\frac{dVm}{dt}_{repol}$) of the action potential (AP) was chosen based on research by Chang et al.^28^, which identified AP with the highest repolarization slope from the last 250 stimulations. Additionally, the maximum slope of the depolarization phase, $\frac{dVm}{dt}_{max}$, is associated with AP’s pulse. Vm resting signifies the resting potential of the AP, while ${\text{APD}}_{50}$ and ${\text{APD}}_{90}$ represent the duration from peak to 50% and 90% repolarization, respectively. ${\text{APD}}_{tri}$ signifies the triangle area between ${\text{APD}}_{90}$ and ${\text{APD}}_{50}$.

Likewise, calcium transient durations for 50% and 90% repolarization, represented by $Ca{D}_{50}$ and $Ca{D}_{90}$, are considered biomarkers (Fig. 2). $C{a}_{tri}$ represents the triangle area between $Ca{D}_{90}$ and $Ca{D}_{50}$, while $C{a}_{Diastole}$ indicates the calcium concentration during the diastolic phase. These biomarkers were strategically chosen to capture a comprehensive range of cellular responses to drug effects, providing robust features for machine learning analysis.

### The grid search for optimizing machine learning models

GS is a preferred method for hyperparameter tuning in machine learning models, systematically exploring various combinations of hyperparameters across a grid and evaluating performance using a validation set^33^. This method systematically explores all combinations of hyperparameter values in a grid, assessing the performance of each variant with a validation set^34^. We allocated 80% of our dataset for training and the remaining 20% for validation, allowing for a rigorous optimization process during GS. The models, including ANN, XGBoost, RF, SVM, K-Nearest Neighbors (KNN), and radial basis function (RBF) networks, underwent fivefold cross-validation within the training subset to enhance their robustness and minimize overfitting. GS iteratively trains on all hyperparameter combinations, selecting the configuration that excels in validation performance^34^. Despite its thoroughness, GS can be computationally intensive as the hyperparameter space expands^35^. Therefore, we constrained the hyperparameter selection to balance computational efficiency and model performance, ultimately applying the best-configured model to evaluate the test set^33^.

ANN are interconnected neural networks consisting of several layers, including input, hidden, and output layers^36^. The proposed ANN model is designed with an input layer representing complex interactions captured by 12 in-silico biomarkers. Each of these biomarkers contains detailed information from interactions with 12 different drugs, where each drug is represented by 2000 data points, culminating in a rich dataset for model training.

The GS method was employed to optimize the ANN configuration, focusing on parameters such as batch size, with 32 and 64 being considered, and optimizer choices, including RMSprop and Adam. Neuron counts in the range of 5–9 and learning rates of 0.1, 0.01, and 0.001 were also explored to fine-tune the model’s performance. The model’s training process included a rigorous fivefold cross-validation, where the dataset was divided into 80% for training and 20% for validation in each fold, ensuring comprehensive and unbiased model assessment. The training utilized a batch size of 32, allowing for efficient iterative learning and weight updating.

**Fig. 3:**
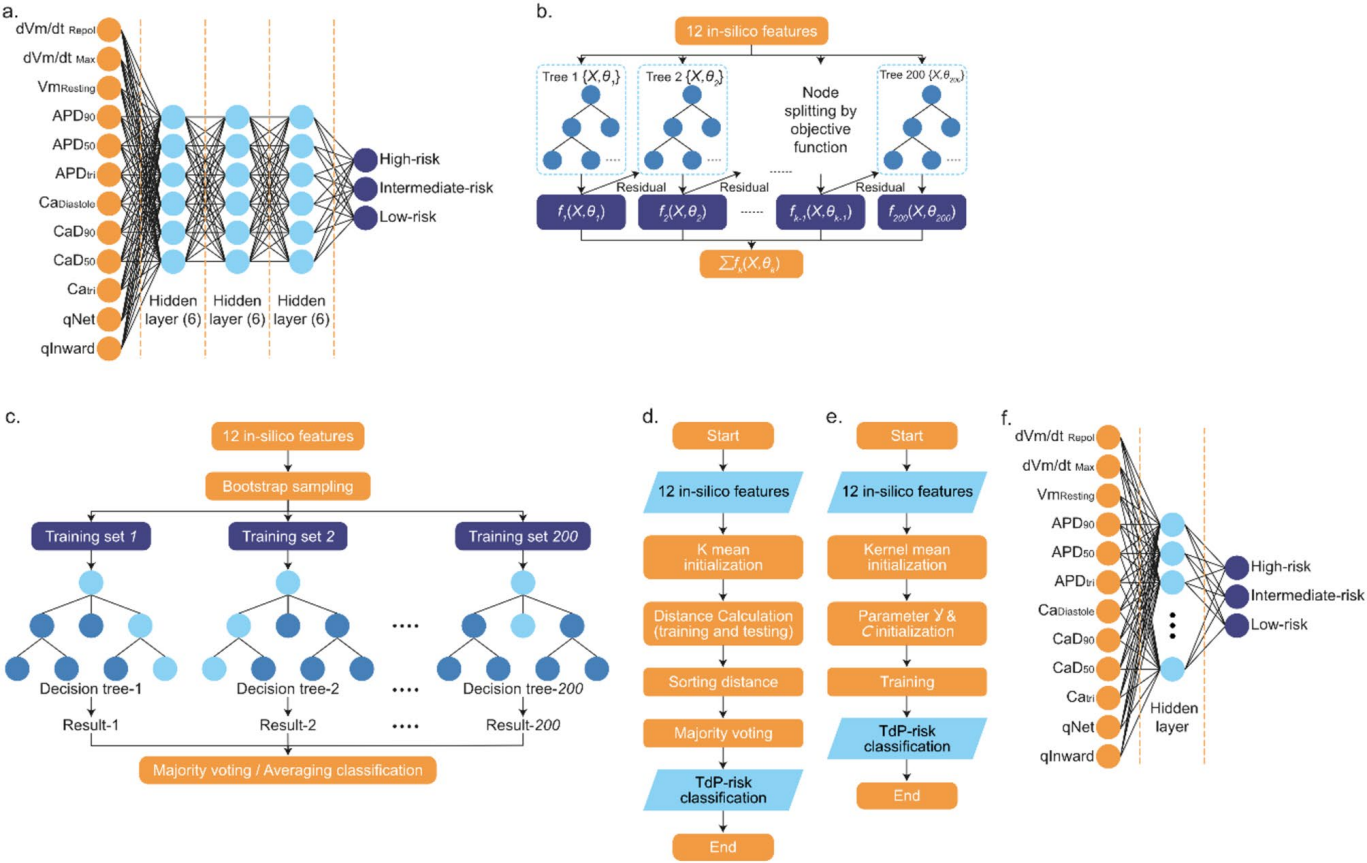
(**a**) Schematic representation of the ANN classification model, which employs twelve in-silico biomarkers as inputs. The model architecture comprises three hidden layers, each consisting of six neurons. Outputs from the ANN model are categorized into three risk classes: high-risk, intermediate-risk, and low-risk for TdP. (**b**) Illustration of the XGBoost classifier model. This model utilizes twelve in-silico features to train an ensemble of 200 decision trees. The trees are built sequentially with each tree learning from the errors (residuals) of the previous ones, thereby refining the classification. The node splitting is guided by an objective function, and the final output is the sum of predictions from all trees. (**c**) Depiction of the Random Forest training process. Starting with twelve in-silico features, the method employs bootstrap sampling to create multiple training sets. Each set is used to train a decision tree, resulting in 200 trees. The classification outcome for a sample is then determined by majority voting or averaging the results from all decision trees. (**d**) Workflow of the KNN classifier. The process begins with twelve in-silico features and initializes using K mean. It calculates the distance between training and testing points, sorts by distance, and applies majority voting for TdP risk classification, resulting in the final output. (**e**) Process diagram for the SVM classifier. The model initiates with twelve in-silico features and uses kernel mean initialization. Parameters Y & C are then established, followed by the training phase, culminating in the TdP risk classification. (**f**) Diagram of the RBF. The network starts with twelve in-silico features leading into a hidden layer that applies the RBF for transformation. The output is divided into three risk categories: high, intermediate, and low risk for TdP.

Before training, the data underwent Z-score normalization to ensure a consistent scale across all inputs, enhancing the model’s classification accuracy^37^. The ANN architecture features three hidden layers, each with six nodes, and an output layer with three nodes (see Fig. 3a). The selection of ReLU (Rectified Linear Activation) as the activation function for hidden layers introduces non-linearity, enhancing model learning without impeding gradient flow. In the output layer, the softmax activation function converts the neural output to a probability distribution, accurately reflecting class probabilities^15^.

The loss function, ‘categorical cross-entropy’, was used to gauge the difference between the predicted and actual probability distributions, guiding the model towards precise predictions. The ‘adam’ optimizer was chosen for its effectiveness in managing sparse gradients and expediting the training process^38^. These methodical design and validation steps were taken to ensure the ANN model effectively interprets the intricate relationships within the biomarker-drug interaction data, leading to reliable drug effect classification.

XGBoost, an advanced gradient boosting decision tree algorithm, excels in constructing efficiently enhanced trees that operate in parallel^39^. It incorporates regularization to optimally balance the reduction of the objective function and model complexity, thus preventing overfitting^40,41^. In our study, the XGBoost model is specifically employed to classify the responses of 12 in-silico biomarkers, each representing interaction information from 12 different drugs (see Fig. 3b). Notably, each of these drugs encompasses 2000 data points, detailing the effects and interactions with the biomarkers, thus providing a comprehensive dataset for the model to analyze and predict the biomarker responses effectively^42^. Figure 3b depicts a schematic representation of XGBoost classification, illustrating how the algorithm iteratively improves its predictions.

During the training process, the model calculates loss for each node to identify the most impactful leaf nodes for the decision-making process. XGBoost dynamically adds new decision trees, segmenting input features to enhance learning precision. This iterative addition aims to refine the model’s predictions by learning new functions ${f}_{k} (X, k)$ that align with the accumulated knowledge. After completing the training, with K decision trees developed, each sample’s prediction traverses these trees, concluding in specific leaf nodes, each assigned a score. The aggregated scores from all trees determine the final predicted efficacy for each biomarker.

Optimizing the XGBoost classification model involved using GS to select optimal parameters. This entailed testing various configurations of the number of trees (50, 100, 150, 200) and maximum depth levels (3, 5, 7, 9), enabling us to find the best balance for model performance. Applying fivefold cross-validation ensured a rigorous assessment of the model’s predictive accuracy, reinforcing its reliability and effectiveness in deciphering complex biomarker-drug interactions.

RF is a practical ensemble learning approach for classification. In this method, a collection of decision trees is constructed during the training process, with the final output obtained through the aggregate estimation of individual trees^43,44^. This model integrates various predictions to produce more accurate and stable results.

In the context of our study, the RF model leverages complex information from 12 in-silico biomarkers (Fig. 3c), each associated with data from 12 different drugs. Furthermore, each drug is represented by a large dataset of 2000 data points, indicating the drug’s response under various conditions and scenarios. In our model, each biomarker interacts with an extensive dataset containing detailed information about the response to each of the 12 drugs. Consequently, the RF model analyzes this data to identify patterns and relationships, enabling accurate predictions about the most suitable drug based on the patient’s biomarker profile.

We applied the GS method to optimize the model, selecting the number of decision trees from 100, 150, and 200, and determining the maximum features with options ‘log2’, ‘auto’, and ‘sqrt’^44–46^. The maximum depth of the trees was set using values ‘None’, 3, 5, 7, and 9, employing entropy and Gini selection criteria to assess the best splits.

During the training process, we implemented fivefold cross-validation to evaluate the model’s performance objectively. The model was trained using 80% of the available data, while the remaining 20% was used to validate and test the accuracy of the model’s predictions.

Our study chose the SVM framework for its precision in handling high-dimensional data (Fig. 3d). We employed StandardScaler to normalize feature vectors, ensuring uniform scale across dimensions and enhancing the model’s statistical robustness^47,48^. StratifiedKFold for cross-validation, divided into five folds, ensured that each subset maintained class distribution homogeneity, which is vital for reliable performance evaluation. Through GS, we optimized hyperparameters, selecting a regularization parameter (C) of 1, a kernel coefficient (gamma) of ‘scale’, and the RBF kernel, balancing model complexity and preventing overfitting.

The optimal settings were deduced to streamline model operations, where StratifiedKFold ensured even class representation in each training subset, and GS systematically explored parameter combinations to enhance model efficacy. This systematic training and validation process, where accuracy scores were diligently recorded across folds, revealed SVM’s adaptability and resilience, reflected in synthesized average accuracy metrics^47–49^. These empirical findings underscore SVM’s competence in deciphering complex patterns within the dataset, affirming its applicability and effectiveness in complex classification tasks within machine learning research.

The KNN algorithm, fundamental in machine learning, classifies data points based on their proximity to their nearest neighbors (Fig. 3e). It operates under the premise that similar items are closer to each other in the feature space. The ‘k’ in KNN refers to the number of nearest neighbors considered for the majority voting process that determines the class of the queried point^47^.

Optimization in this study was carried out using GS, focusing on key parameters: the number of neighbors (n_neighbors) and the distance metric (Euclidean or Manhattan). The number of neighbors affects the granularity of the classification, with too few neighbors leading to noise sensitivity and too many causing underfitting. The distance metric determines how similarity is calculated, influencing the grouping and classification outcome.

A StratifiedKFold cross-validation approach was employed to ensure each class was evenly represented across folds, thus maintaining validity and reliability in the model’s performance assessment. Through GS, we identified the optimal hyperparameters, which were then applied to construct and evaluate the refined KNN model. The process involved systematic iteration over different data subsets and assessing the model’s performance in terms of accuracy in each fold.

To illustrate, imagine a two-dimensional feature space where data points from two classes are spread. KNN classifies a new point based on the majority class within its ‘k’ nearest neighbors, with distance measured in either a straight line (Euclidean) or grid-like path (Manhattan).

The empirical analysis revealed that the KNN model exhibited robustness and high accuracy with the optimized settings, signifying its capability to discern complex patterns. Compared to other classification methods, the inherent adaptability and straightforward logic of KNN make it a reliable choice for various practical applications.

RBF networks are artificial neural networks that are known for their ability to handle nonlinear relationships^50^. They are commonly used in machine learning for pattern recognition and classification tasks. RBF networks transform the input space into a higher-dimensional space using RBF, typically Gaussian, to facilitate complex classification scenarios^50–52^.

In our study, the RBF network implemented within the Sequential neural network model utilizes TensorFlow and Keras. The key parameters of this RBF layer include the ‘units’, representing the number of neurons, and ‘gamma’, a scale factor that influences the spread of the radial basis function, affecting how the network discriminates between different input features (Fig. 3f).

During initialization, the centers of the RBF neurons are randomly set with a uniform distribution to diversify the starting points for learning. Operationally, the RBF layer computes its output by applying the Gaussian function to measure the Euclidean distance between inputs and these centers, adjusted by the ‘gamma’ parameter^53,54^. This mechanism enables the network to project the input into a higher-dimensional space, enhancing classification capabilities.

The network performance was optimized using GS, a systematic approach to parameter optimization. This process, integrated seamlessly within Keras via the KerasClassifier wrapper, allows for efficient exploration across the specified parameter grid, ensuring the most effective combination for model training is identified. Following the RBF layer, the model includes a dense layer with softmax activation to classify the transformed inputs into three distinct categories, meeting the multi-class classification requirements. Rigorous fivefold stratified cross-validation was employed during training to assess the model’s reliability and consistency across different data subsets.

By adopting these methods, we leveraged the unique qualities of RBF networks, renowned for their nonlinear handling and robustness in machine learning tasks. Integration with advanced deep learning frameworks like TensorFlow and Keras enhanced functionality and usability and allowed for extensive experimentation and detailed performance analysis. Theoretical strengths of RBF combined with practical, scalable deep learning practices ensured a comprehensive approach to tackling the complexities of sophisticated classification scenarios.

This study showcases the RBF network’s effectiveness in practical classification problems, offering insights into its performance relative to other networks or traditional classification methods. The practical implications of utilizing RBF networks in machine learning are vast, demonstrating their value and relevance across various industries, particularly where complex nonlinear patterns must be deciphered and classified accurately.

### SHAP (SHapley Additive exPlainations)

In this study, the role of XAI, particularly the SHAP method, is crucial in understanding the factors driving machine predictions^27^. Unlike traditional approaches focusing solely on improving model performance through dimensionality reduction, our goal with XAI is to enhance model performance and interpretability simultaneously. The SHAP method enables us to explore physiological insights related to each biomarker, providing a detailed breakdown of the contribution of each biomarker to predictions without sacrificing information richness^27^. The strategic use of XAI aligns with our objective to maintain the complexity of the analysis process while gaining an in-depth understanding of the underlying factors^27^. We also consider the research methodology employed by Li et al.^55^, which applied a single feature without reducing its dimensionality, although not explicitly utilizing XAI. By adopting the SHAP method, we go beyond conventional approaches, ensuring that the interpretability of our model is not compromised during the performance optimization process^26^. The analogous to fair payments for contributions in a game, SHAP values allow us to understand the average marginal contribution of feature values, providing a comprehensive perspective on complex relationships within the dataset^56^. SHAP value can be calculated using Eq. (5)^19^.

5 $${\varnothing }_{i}\left(N,v\right)=\frac{1}{N!}\sum_{S\subseteq N\{i\}}\left|S\right|!\left(N-\left|S\right|-1\right)!\left[{V}_{S\cup \left\{i\right\}}\left({x}_{S\cup \left\{i\right\}}\right)-{V}_{S}\left({x}_{S}\right)\right]$$

${\varnothing }_{i}$ is the contribution of the i_*-th*_ feature, N is the number of features, S is the subset of the features used in the model, and x is the feature value vector of the instance to be described. $\left[{V}_{S\cup i}\left({x}_{S\cup i}\right)-{V}_{S}\left({x}_{S}\right)\right]$ is the prediction for feature values in the marginalized set S of features not included in the S set. The model ${\text{V}}_{\text{S}\cup \text{i}}$ is trained with the presence of the i-th feature, while another model ${\text{V}}_{\text{S}}$ is trained by omitting the same feature^19^.

### Feature importance

The SHAP feature importance is the sum of the average absolute Shapley values for each feature across all data^56^. The sum of Shapley’s scores is absolute because Shapley’s values positively and negatively affect predictive results in machine learning^57^. The importance of the SHAP feature can be represented by Eq. (6)^56^.

6 $${I}_{i}=\frac{1}{n}\sum_{j=1}^{n}\left|{\varnothing }_{i}^{\left(i\right)}\right|$$

${\varnothing }_{i}$ is the contribution of the i_*-th*_ feature, $n$ is the number of shapley value. SHAP feature importance differs from permutation feature importance because SHAP is based on the magnitude of the feature attribution, while permutation feature importance is based on decreasing model performance^56^.

### Performance evaluation

**Fig. 4:**
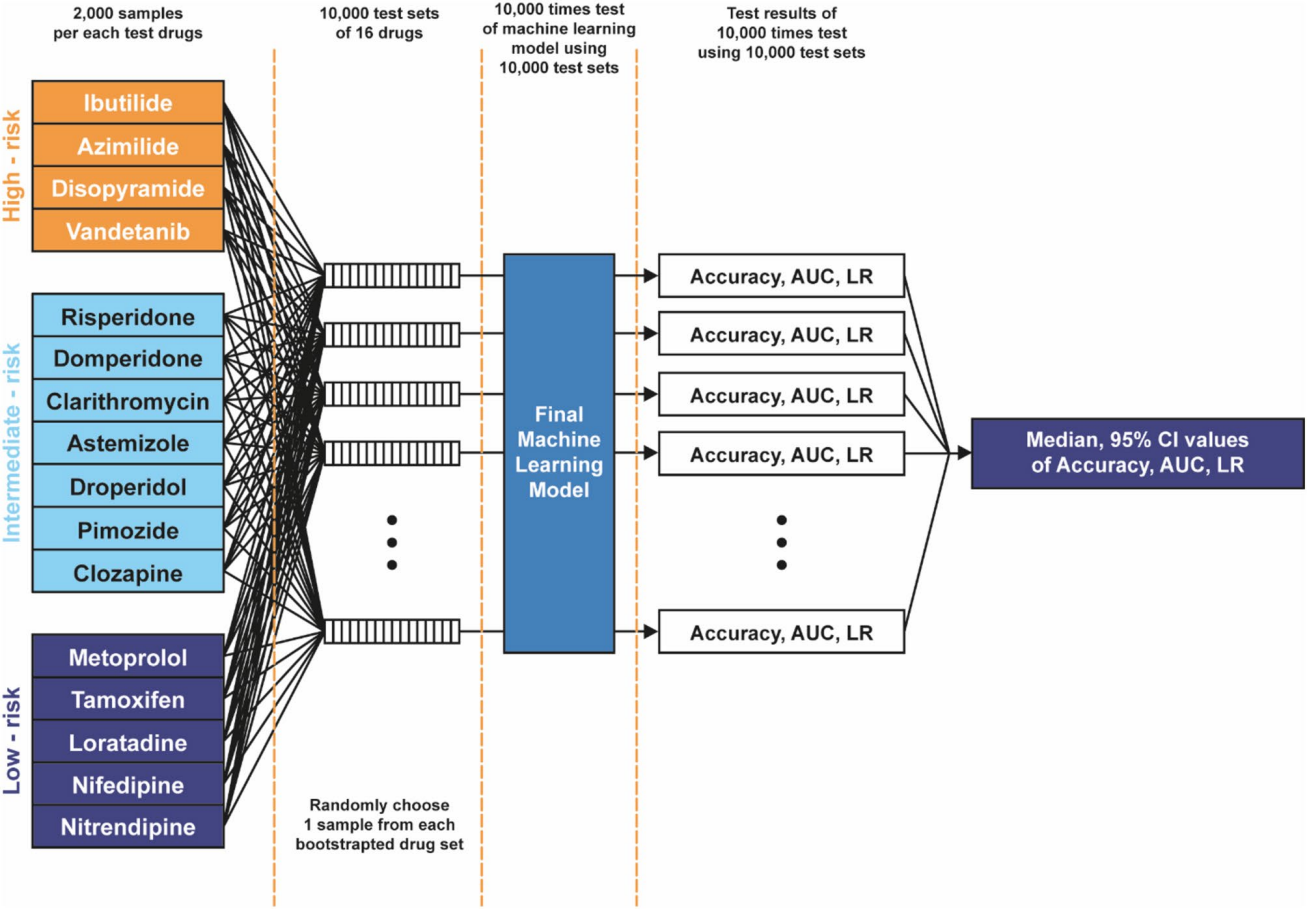
An evaluation algorithm was employed to assess the performance of the classification model proposed by the CiPA research group, utilizing the principles of the central limit theorem; AUC, the area under the receiver operating curve; LR, likelihood ratio.

**Fig. 5:**
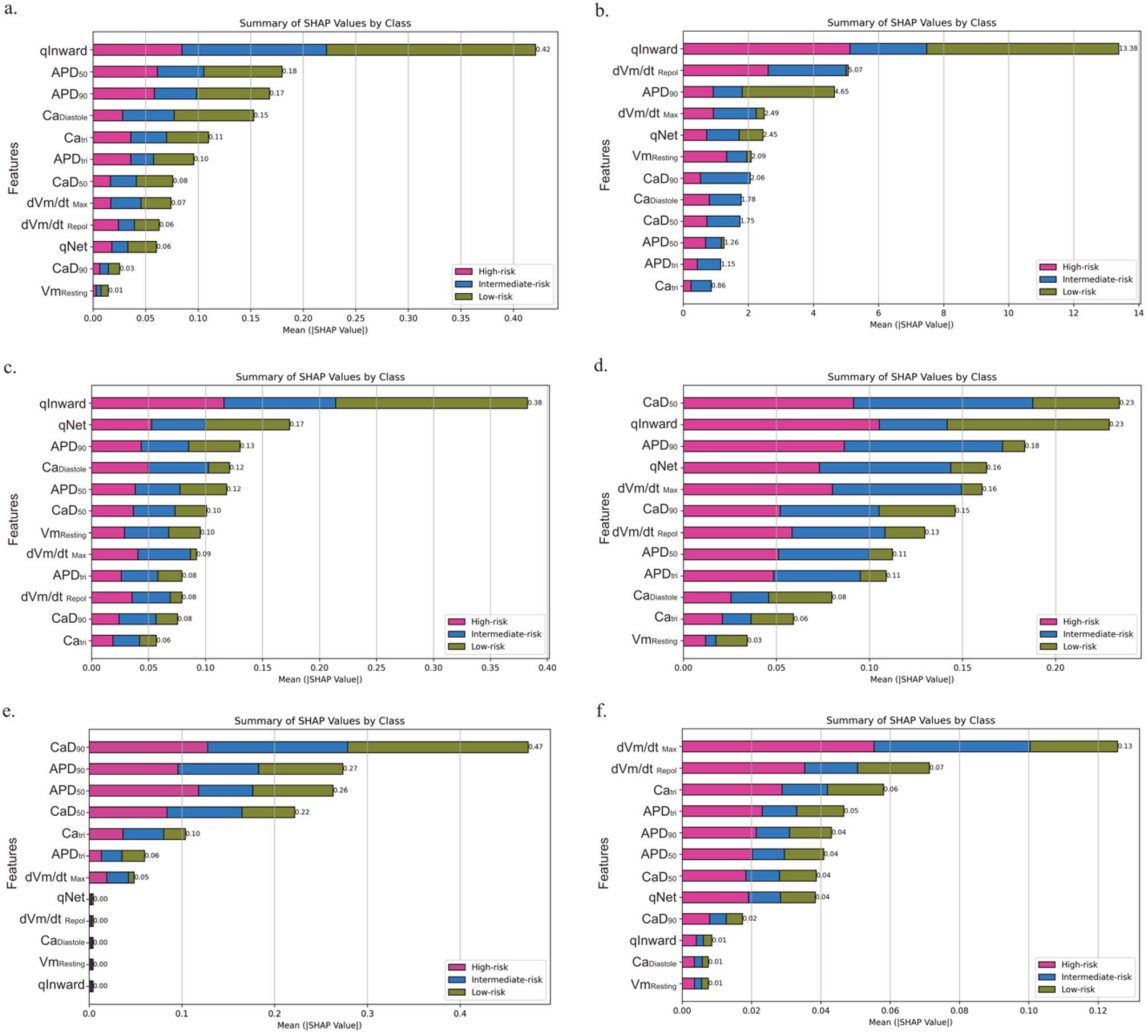
(**a**) Feature importance visualization for the ANN model. The bar chart displays the mean SHAP values of each in-silico biomarker across three classes: high-risk, intermediate-risk, and low-risk. The feature qInward appears to be the most influential for high-risk classification, while Vm Resting has the least impact. (**b**) Feature importance chart for the XGBoost model. The graph illustrates the average impact of each in-silico biomarker on the model’s output, with higher mean SHAP values indicating greater importance. For high-risk classification, qInward and dVmdt Repol show significant influence. (**c**) Summary of SHAP values by class for the RF model. This bar chart represents the mean SHAP values by class, highlighting the features that most strongly affect the model’s predictions, with qInward showing a high impact on the high-risk class. (**d**) SHAP value summary for the SVM model. The bar chart details the feature importance, where CaD_50 is notably influential across all risk categories, suggesting a critical role in the model’s risk stratification process. (**e**) Feature importance for the KNN model, depicted through mean SHAP values. CaD_50 and APD_90 stand out as key features with high importance for the high-risk and intermediate-risk classifications, respectively. (**f**) Visualization of feature importance for the RBF model. The chart highlights the mean SHAP values with dVmdt Max showing a prominent role in distinguishing across all of risk category.

Following the validation procedure implemented by the CiPA research group, we established a drug allocation strategy where 12 drugs were designated for the training set, and 16 drugs were reserved for the testing set (Table 1)^55^. Our study involved the creation of 10,000 drug sets, each consisting of 2000 samples, to ensure a diverse and representative dataset (see Fig. 4). This process included the random selection of drug samples to form these sets. Notably, each drug set comprised one sample from each of the 16 drugs, contributing to the variability and randomness in our dataset. This randomization approach was adopted to prevent potential systematic bias from a predetermined allocation. The 10,000 drug sets are not individual datasets but a collection from which random samples are drawn for each iteration of our analysis. The dataset size was chosen to enhance statistical robustness and facilitate a comprehensive evaluation using a pre-trained machine learning in 10,000 classification tests. The results of these tests, meticulously documented and presented in Fig. 5, underwent detailed analysis, including examining median values and 95% confidence intervals (CI). The 95% CI was calculated as the 2.5th to 97.5th percentiles of measurements across the 10,000 datasets for each observed test parameter, following the methodology proposed by Li et al.^55^. This comprehensive approach addresses dataset size concerns and ensures our classification models’ reliability and robustness under diverse conditions, providing valuable insights into their performance. Model performance testing uses Eqs. (7–11)^29,58–60^.

7 $$Accuracy=\frac{TP+TN}{TP+FP+TN+FN}$$

8 $$Sensitivity \left(Recall\right)=\frac{TP}{TP+FN}$$

9 $$Specificity=\frac{TN}{TN+FP}$$

10 $$LR+=\frac{Sensitivity}{1-Specificity}$$

11 $$LR-=\frac{1-Sensitivity}{Specificity}$$

In this study, accuracy refers to the correctness of positive sample classifications, while recall (sensitivity) quantifies the proportion of correctly identified positive predictions among all actual positive instances^29,58,59,61^. Specificity, on the other hand, gauges the model’s ability to identify true negative instances correctly. It is crucial to note that LR stands for Likelihood Ratio. We employed recall (sensitivity) and specificity to compute the Positive Likelihood Ratio (LR+) and Negative Likelihood Ratio (LR−), following the methodology applied by CiPA and previous studies, such as those conducted by Yoo et al., Li et al. 2019 and Yoo et al.^15,29,55^.

LR+ provides the probability of a positive outcome after a positive screening, while LR− offers the probability of a negative outcome after a negative screening^29,61^. True Positive (TP) characterizes the quantity of data accurately predicted as positive, whereas False Negative (FN) indicates instances where the model predicts a negative class when it should be positive. False Positive (FP) occurs when data that should be negative is incorrectly predicted as positive. Conversely, True Negative (TN) reflects the quantity of data correctly predicted as negative^60^.

It is essential to note that we did not present the values of sensitivity and specificity in this report, aligning with the focus of our research, which follows the methodology of previous studies by Yoo et al., Li et al. and Yoo et al.^15,29,55^. These studies also solely presented AUC, LR+, and LR− values. Similarly, we adopted a comparable approach to facilitate a straightforward comparison of results with these prior research endeavors.

## Results

### Deciphering the role of electrophysiological biomarkers in drug toxicity prediction: a SHAP-based comparative analysis of machine learning models

This study delved into the impact of electrophysiological biomarkers on drug toxicity prediction, employing SHAP-based analysis to comprehend the specific feature contributions within various machine learning models^27^. SHAP, an advanced model interpretation method, provides measurable insights into each biomarker’s influence on model predictions, facilitating a deep understanding of how electrophysiological characteristics affect drug toxicity risk^57^. In the ANN context (Fig. 5a), SHAP analysis revealed significant contributions from biomarkers such as qInward with a SHAP value of 0.42, indicating its primary role in the predictive mechanism, followed by $AP{D}_{50}$ at 0.18, $AP{D}_{90}$ at 0.17, and $C{a}_{Diastole}$ at 0.15. These findings highlight the ANN’s sensitivity to ionic fluctuations and action potential duration variations. Meanwhile, the XGBoost model placed the highest emphasis on qInward with a SHAP score of 13.38 (Fig. 5b), underscoring the importance of cellular dynamics, with subsequent notable values for $\frac{dVm}{dt}_{repol}$ at 5.07, $AP{D}_{90}$ at 4.65, and $\frac{dVm}{dt}_{max}$ at 2.49, illustrating the model’s focus on depolarization and repolarization processes. The RF model demonstrated a balanced approach in feature importance (Fig. 5c), with qInward and qNet each garnering a SHAP value of 0.23, illustrating how RF integrates diverse electrophysiological signals for robust prediction. Conversely, the SVM highlighted $Ca{D}_{50}$ with a SHAP value of 0.32 (Fig. 5d), while the KNN model prioritized $Ca{D}_{90}$ with a SHAP score of 0.49, emphasizing the significance of calcium duration in their analysis (Fig. 5e). The RBF model spotlighted $\frac{dVm}{dt}_{max}$ with a SHAP value of 0.13, accentuating its focus on transient electrophysiological dynamics (Fig. 5f). Through SHAP analysis, this study provides nuanced understanding of how each machine learning model processes and evaluates electrophysiological information, underlining the importance of a multidisciplinary approach in drug toxicity analysis. These results not only advance our comprehension of electrophysiology and machine learning but also contribute to the development of more informative and effective drug toxicity risk assessment methods, offering valuable insights for future drug toxicity prediction strategies.

### SHAP value distributions across machine learning models in biomarkers

In the Supplementary Material, Fig. S1a–r meticulously illustrate SHAP (SHapley Additive exPlanations) value distributions across six classification models, offering a granular view of how individual features contribute to each model’s predictive power. SHAP values, a concept central to explainable AI, serve to quantify the effect of each feature on the model’s predictions, delineating their importance in the predictive process. These values are stratified into high, intermediate, and low impact categories to facilitate an in-depth analysis of the feature importance hierarchy within each model, correlating directly with their statistical significance and predictive impact.

Figure S1a–c in the supplementary material correspond to the ANN, depicting the SHAP value distribution. Figure S1 a illustrates high-impact features at high-risk levels such as qInward and ${APD}_{90}$, Fig. S1b shows high-impact features at intermediate-risk levels including qInward and ${Ca}_{Diastole}$, while Fig. S1c represents high-impact features at low-risk levels like qInward and ${Ca}_{Diastole}$. The gradation in feature importance highlights the role of electrophysiological parameters within the ANN analytical framework.

For the XGBoost model, Fig. S1d–f in the supplementary material articulate the tiered feature influence, with Fig. S1d emphasizing high-impact features at high-risk levels such as qInward and $\frac{dVm}{dt}_{repol}$, Fig. S1e showing high-impact features at intermediate-risk levels like $\frac{dVm}{dt}_{repol}$ and qInward, and Fig. S1f depicting high-impact features at low-risk levels like qInward and ${APD}_{90}$. These figures collectively underline the strategic assessment of the model towards dynamic cellular properties.

The significance of features in the RF model is illustrated through Fig. S1g–i in the supplementary material. Figure S1g highlights high-impact features at high-risk levels including qInward and qNet, Fig. S1h displays high-impact features at intermediate-risk levels like qInward and ${Ca}_{Diastole}$, and Fig. S1i identifies high-impact features at low-risk levels like $\frac{dVm}{dt}_{repol}$ and $\frac{dVm}{dt}_{max}$. This demonstrates the RF model’s comprehensive approach to integrating a wide spectrum of electrophysiological features.

The SVM model analysis is encapsulated in Fig. S1j–l of the supplementary material. High-impact features at high-risk levels like ${CaD}_{50}$ and qInward are presented in Fig. S1j, high-impact features at intermediate-risk levels like ${CaD}_{50}$ and ${APD}_{90}$ in Fig. S1k, and high-impact features at low-risk levels including qInward and ${CaD}_{50}$ in Fig. S1l. This distribution reflects the SVM’s methodical consideration of feature weights in its decision-making process.

In the context of the KNN model, Fig. S1m–o in the supplementary material illustrate the SHAP value distribution, with Fig. S1 m depicting high-impact features at high-risk levels like qInward and ${APD}_{50}$, Fig. S1n showing intermediary features like ${CaD}_{50}$ and ${APD}_{90}$, and Fig. S1o outlining high-impact features at low-risk levels like ${CaD}_{50}$ and ${APD}_{90}$. This highlights the KNN model’s reliance on the local significance of electrophysiological attributes for classification accuracy.

Lastly, the feature priorities of the RBF network are presented in Fig. S1p–r of the supplementary material, with Fig. S1p depicting high-impact features at high-risk levels like $\frac{dVm}{dt}_{max}$ and $\frac{dVm}{dt}_{repol}$, Fig. S1q encompassing high-impact features at intermediate-risk levels including $\frac{dVm}{dt}_{max}$ and $\frac{dVm}{dt}_{repol}$, and Fig. S1r illustrating high-impact features at low-risk levels like $\frac{dVm}{dt}_{max}$ and $\frac{dVm}{dt}_{repol}$. This emphasizes the RBF model’s attention to key features that determine kernel-based similarity assessment.

The elaborate portrayal of SHAP value distribution in the supplementary material provides an extensive explanation of the feature-based discriminative power of each model, underscoring the complexity and specificity of feature importance in the context of predictive modeling. This granular analysis enhances our understanding of the decision-making paradigms of the models, offering substantial insights into the intricate dynamics that govern machine learning in electrophysiological data interpretation.

### Quantitative analysis of feature impact on model predictions using SHAP

In this study, we delve into the intricate landscape of feature influence on model predictions by employing SHAP (SHapley Additive exPlanations) analysis, a cornerstone in the field of explainable AI. SHAP values are used to quantify the impact of individual features on a model’s predictions, providing a clear and interpretable measure of each feature’s relative importance. These values play a crucial role in elucidating the mechanisms behind model decision-making, thus enhancing transparency and understanding. Illustrated in the supplementary material, the waterfall plots categorize the impact of features into high, intermediate, and low levels across six classification models. These plots systematically depict the incremental contribution of each feature, offering a detailed view of how specific features influence the prediction outcomes in these models. Through this analysis, we gain a nuanced understanding of the dynamic interplay between features and their roles in predictive modeling.

In the Supplementary Material, the waterfall plots for the Artificial Neural Networks (ANN) model (Fig. S2a–c) distinctly delineate the feature hierarchy and its influence on model predictions. In these plots, risk levels are demarcated to demonstrate how features contribute differently under varying conditions of predictive uncertainty. Figure S2a, representing the high-risk category, exhibits features like ${APD}_{50}$ and ${APD}_{90}$ as having substantial contributions, signifying their pivotal roles when the prediction stakes are high and the model’s output is most critical. These features, due to their strong impact, are likely to drive significant shifts in model prediction outcomes under scenarios classified as high-risk. Figure S2b transitions to the intermediate risk level, where features like $\frac{dVm}{dt}_{max}$ and ${Ca}_{tri}$ are shown. Although these features have a noteworthy impact, their influence is moderated compared to the high-risk features, indicating their balanced role in scenarios where predictions carry a moderate level of certainty and risk. This intermediate category bridges the high and low-risk features, showing a gradient of feature impact on the model’s predictions. Lastly, Fig. S2c captures the low-risk features such as ${CaD}_{50}$ and ${Ca}_{Diastole}$, which, despite their influence on the model, contribute less decisively compared to the high and intermediate-risk features. Their presence in the low-risk category highlights their subtle yet consistent role in shaping the model’s output when the predictions are relatively stable or less prone to change.

In the XGBoost analysis, the supplementary material’s Fig. S2d–f elucidate the SHAP value distribution and its correlation with different risk levels, providing a clear depiction of how features impact the model’s predictions. Figure S2d, representing the high-risk category, underscores the prominent influence of features like qInward and qNet, which are critical in driving the model’s high-stakes predictions. These features, with their substantial SHAP values, are deemed high-impact due to their strong predictive power in scenarios where the potential for drug-induced toxicity is greatest. Moving to the intermediate risk level, Fig. S2e showcases features such as ${CaD}_{90}$ and ${Ca}_{tri}$, which, while still influential, have a moderated effect on the model’s output compared to the high-risk features. This intermediate category bridges the gap between the most and least influential features, representing conditions of moderate predictive uncertainty where these features still play a significant but less decisive role. At the low-risk level, Fig. S2f focuses on features like ${APD}_{50}$ and ${Ca}_{tri}$, which have lower SHAP values, indicating their lesser, yet still relevant, contribution to the model’s prediction capabilities. These low-impact features are more subtle in their influence, affecting predictions where the likelihood of toxicity is lower, and thus the predictive certainty is higher.

RF model, leveraging SHAP Waterfall plots presented in Fig. S2g–i. These plots elucidate the incremental influence of each feature compared to a baseline, clarifying their roles in shaping the model’s predictions for specific instances. Figure S2g focuses on high-risk scenarios, demonstrating that features like qInward and qNet possess significant positive SHAP values, enhancing the model’s predictive likelihood, while negative values would indicate a diminishing effect on predictions. This suggests that qInward and qNet are pivotal in escalating the model’s risk assessment, underlining their substantial positive impact on the prediction outcome. In Fig. S2h, which represents intermediate-risk situations, the waterfall plot reveals the impact of features such as $\frac{dVm}{dt}_{repol}$ and ${CaD}_{50}$, alongside others. Here, $\frac{dVm}{dt}_{repol}$ and ${CaD}_{50}$ emerge as influential, with their considerable positive contributions marking them as integral in deciphering the model’s decision-making process. This nuanced representation helps in understanding the intermediate-risk features’ incremental effects on model behavior. Finally, Fig. S2i reiterates the analysis for low-risk levels, aligning with the observations in Fig. S2g but potentially differing in the impact magnitude of features like $AP{D}_{50}$ and $AP{D}_{90}$. This consistency in feature presence across risk levels, with variable impact magnitudes, accentuates the nuanced role these features play in the RF model’s predictive logic, with $AP{D}_{50}$ and $AP{D}_{90}$ notably shaping the prediction outcomes.

In the results section, the SHAP Waterfall plot analysis for the Support Vector Machine (SVM) model is elaborated, showing how variably different features contribute to the predictive outcomes, with Fig. S2j–l in the supplementary material ranking these features based on their importance and contribution to SVM’s decision-making. Specifically, in Fig. S2j, high-impact features like qInward, $C{a}_{Diastole}$, and $C{a}_{tri}$ are highlighted at the top, signifying their significant influence on the model’s predictions, indicative of high-risk scenarios, whereas lower-ranked features such as $Ca{D}_{50}$ and $\frac{dVm}{dt}_{repol}$ suggest a lesser impact, associated with lower risk levels in the model’s evaluative process. Moving to Fig. S2k, a notable reshuffling shows $Ca{D}_{50}$, qNet, and $Ca{D}_{90}$ rising in importance, demonstrating their strong influence and revealing the dynamic nature of feature prioritization in correspondence to intermediate risk levels of SVM’s analytical phase. Finally, Fig. S2l showcases qInward, $Ca{D}_{50}$, and $Ca{D}_{90}$ leading the feature list, asserting their dominant role in molding the model’s output, while features like $\frac{dVm}{dt}_{max}$ and $AP{D}_{50}$, though contributing significantly, are positioned lower, underlining a nuanced hierarchy where the interplay of features shifts based on their assessed impact in influencing the model’s predictions at different risk levels, thereby providing a comprehensive view into how SVM navigates and weighs electrophysiological features in its predictive logic, correlating feature significance directly with the stratified risk assessment in its analytical framework.

In the k-Nearest Neighbors (KNN) model’s SHAP Waterfall plot analysis, represented in Fig. S2m–o of the supplementary material, we delve into the nuanced understanding of how individual features assert their influence on predictive outcomes. High-impact features like $Ca{D}_{90}$ and $AP{D}_{90}$ are prominently displayed at the top in Fig. S2m, signaling their significant roles in high-risk prediction scenarios, while mid-tier and lower-tier features like $V{m}_{Resting}$ and $\frac{dVm}{dt}_{repol}$ are positioned to reflect their moderate and lesser degrees of influence, respectively. This structured hierarchy not only delineates the gradation of feature impacts but also correlates with the model’s risk-level assessment, showcasing a clear differentiation in feature prioritization. As we transition to Fig. S2n, the consistent prominence of $Ca{D}_{90}$ and $AP{D}_{90}$, coupled with $AP{D}_{50}$ and $C{a}_{tri}$, illustrates a dynamic adjustment in the model’s sensitivity, indicating a shift in feature importance across different risk levels. Figure S2o further emphasizes this trend, with $Ca{D}_{90}$ and $AP{D}_{50}$ leading the influence chart, accentuating their pivotal effects on the model’s low-risk predictions. The subsequent ranking of features like $AP{D}_{90}$, $AP{D}_{tri}$, $Ca{D}_{50}$, and $\frac{dVm}{dt}_{max}$ unfolds the intricate interplay within the predictive process, highlighting how these elements collectively fine-tune the KNN model’s accuracy across a spectrum of risk considerations. This in-depth analysis encapsulates the KNN model’s analytical depth, providing a comprehensive view of how electrophysiological features are intricately woven into its decision-making fabric, thereby elucidating the sophisticated machinery driving its predictive mechanics in the realm of electrophysiological data analysis.

The SHAP Waterfall plots for the RBF model, as shown in Fig. S2p–r of the supplementary material, offer a detailed exposition of how specific features impact the model’s predictive behavior across varying risk levels. In Fig. S2p, high-risk features such as $\frac{dVm}{dt}_{repol}$, $AP{D}_{90}$, and $\frac{dVm}{dt}_{max}$ are identified as having a significant influence on the model’s decisions, underscoring their critical role in shaping high-risk predictions and denoting their dominant impact in the model’s analytical hierarchy. Transitioning to Fig. S2q, the analysis shifts to an intermediate-risk perspective, where the same features like $\frac{dVm}{dt}_{repol}$ and $\frac{dVm}{dt}_{max}$ continue to exhibit importance but in a varied context, reflecting the model’s adaptability and nuanced response to the dynamic electrophysiological landscape. This shift underscores the variability and sensitivity of the RBF model to these key features, demonstrating their adjusted significance under different risk assessments. In the low-risk spectrum, depicted in Fig. S2r, there is a further delineation of feature impact, with $AP{D}_{50}$ and $Ca{D}_{50}$ coming to the forefront alongside the persistent influence of $\frac{dVm}{dt}_{repol}$ and $AP{D}_{90}$, which elucidates their ongoing, albeit less pronounced, role in influencing the model’s predictions at this risk level. Collectively, these plots methodically illustrate the hierarchical and stratified nature of feature influence within the RBF model, providing a comprehensive view of how it discerns and integrates various electrophysiological features to refine its predictive outcomes across a spectrum of risk levels, thus enriching our understanding of the intricate decision-making processes embedded within this sophisticated machine learning model.

### Comprehensive analysis of machine learning models for enhanced torsades de pointes risk stratification

In this study, we embarked on a detailed exploration of Torsades de Pointes (TdP) risk classification using a refined ANN model. This method integrated grid search-optimized parameters and SHAP-derived feature importance assessments, testing a dataset of 16 drugs with 12 in-silico biomarkers. The rigorous testing process involved 10,000 classification iterations to ensure the reliability of our findings.

Our ANN model underwent extensive hyperparameter optimization, leading to an ideal configuration that yielded a high degree of accuracy—0.83 (CI 0.75–0.83)—and impressive AUC scores for categorizing high-risk (0.96, CI 0.92–1.00), intermediate, and low-risk TdP cases, as seen in Table 2. A deeper analysis indicated that the removal of the Vmresting biomarker significantly enhanced the model’s performance, illustrating the delicate interplay between feature selection and model accuracy.

**Table 2:** The performance of the ANN algorithm is presented after undergoing 10,000 tests on 16 drugs, displaying the median and 95% CI values derived from the 10,000 test outcomes.

System performance parameter	ANN (11 features)	XGBoost (6 features)	RF (6 features)	KNN (6 features)	SVM (7 features)	RBF (6 features)	Yoo et al.^15^	Li et al.^55^
Accuracy	0.83 (0.79, 0.83)	0.67 (0.58, 0.75)	0.71 (0.62, 0.79)	0.75 (0.62, 0.79)	0.67 (0.62, 0.75)	0.71 (0.62, 0.75)	–	–
AUC high risk	0.92 (0.88, 0.96)	0.81 (0.54, 0.94)	0.83 (0.71, 0.94)	0.88 (0.67, 0.99)	0.75 (0.6, 0.88)	0.92 (0.85, 0.94)	0.92 (0.85, 0.96)	0.86 (0.81, 0.9)
AUC intermediate risk	0.83 (0.73, 0.9)	0.56 (0.43, 0.68)	0.56 (0.46, 0.66)	0.6 (0.48, 0.69)	0.73 (0.65, 0.79)	0.71 (0.67, 0.76)	0.83 (0.73, 0.91)	–
AUC low risk	0.98 (0.95, 0.98)	0.67 (0.58, 0.8)	0.74 (0.64, 0.78)	0.71 (0.59, 0.79)	0.46 (0.38, 0.62)	0.75 (0.69, 0.78)	0.98 (0.91, 1)	0.86 (0.82, 0.9)
LR + high risk	500,000.97 (250,001.07, 500,001.98)	3.0 (1.0, 6.0)	3.0 (2.0, 6.0)	9.0 (3.0, nan)	3.0 (1.0, 9.0)	3.0 (2.0, 4.0)	5000 (4000, 6000)	5 (3.33, 12.5)
LR + intermediate risk	2.25 (1.8, 2.25)	1.29 (0.43, 5.14)	1.93 (0.86, 5.14)	1.71 (0.86, 2.57)	1.61 (1.29, 2.57)	inf (0.0, nan)	2.249 (1.80, 2.25)	–
LR + low risk	600,000.92 (400,000.53, 600,002.16)	3.3 (2.2, 4.4)	3.3 (2.2, 4.4)	3.3 (1.1, 4.4)	1.1 (1.1, 2.2)	2.93 (1.65, 6.6)	6000(4.39, 6000)	2.01 (1.61, 2.84)
LR- high risk	0.5 (0.5, 0.75)	0.38 (0.0, 1.0)	0.3 (0.0, 0.67)	0.27 (0.0, 0.6)	0.6 (0.3, 1.0)	0.0 (0.0, 0.0)	0.5 (0.4, 0.59)	0.556 (0.278, 0.588)
LR- intermediate risk	0.0 (0.0, 0.0)	0.86 (0.55, 1.29)	0.73 (0.48, 1.07)	0.64 (0.48, 1.07)	0.32 (0.21, 0.77)	0.86 (0.71, 1.12)	0.18e−3 (0.18e−3, 0.26)	–
LR- low risk	0.4 (0.4, 0.6)	0.49 (0.24, 0.55)	0.49 (0.24, 0.55)	0.49 (0.24, 0.98)	0.98 (0.88, 0.98)	0.27 (0.24, 0.63)	0.4 (0.4, 0.66)	0.118 (1.8e−6, 0.284)

Furthermore, we expanded our analysis to include other classification models for comparison. The XGBoost and RF models, with their respective feature sets, displayed strong predictive abilities, particularly in low-risk classifications. Their accuracies, while not surpassing the ANN, offer valuable insights and underscore the utility of ensemble methods.

The KNN classifier demonstrated its strength in identifying high-risk cases with an AUC of 0.88 (CI 0.67–0.99) (Table 2), highlighting its potential for rapid risk assessment in clinical settings. Similarly, the SVM, with fewer features, achieved an excellent AUC for low-risk classifications (0.98, CI 0.88–0.98), challenging the performance of the ANN (Table 2).

The RBF model, another deep learning-based classifier in our study, showed promising results with a solid performance across different risk levels. Its accuracy and AUC for the intermediate-risk group were competitive, showing its capacity for complex data separation.

Compared to previous studies, our results present significant advancements in TdP risk classification. The ANN model’s positive and negative likelihood ratios for various risk levels demonstrate a high level of accuracy, especially for the high and low-risk categories. Even with the intermediate-risk level’s challenges, our model outperformed existing models, indicating its strength as a reliable clinical tool for risk assessment. These findings not only contribute to the ongoing dialogue in the field but also provide a comprehensive view of the potential of machine learning in clinical decision-making.

### Assessing the impact of feature reduction on predictive model performance

In the results section of our study, we delineate the intricate interdependency between the quantity of features and the efficacy yielded from various predictive models, with a special emphasis on the pivotal role of SHAP values in guiding our feature reduction strategy.

For ANN, reducing the number of features from twelve to eleven had a negligible impact on the ability to predict high-risk cases, with the Area Under the Curve (AUC) remaining stable at 0.87, and the p-value shifting from 0.001364 to 0.006442 (Table 3). This feature reduction paradoxically coincided with peak performance for predicting intermediate and low-risk scenarios, evidenced by AUC values of 0.66 (p = 0.006442) and 0.83 (p = 0.0006442), respectively (Table 3). Such outcomes reinforce the hypothesis that an optimally filtered feature set, selected based on its influence, can surpass a more extensive feature array.

**Table 3:** Impact of feature reduction on model performance metrics—this table presents a factorial ANOVA analysis delineating the effect of systematic feature reduction on the Area Under the Curve (AUC) for high, intermediate, and low-risk predictions across multiple classification models. Reduction in the number of features is directed by SHapley Additive exPlanations (SHAP) value significance. Each entry corresponds to an AUC value, accompanied by the CI in parentheses and the p-value, providing a statistical testament to the performance shifts observed as the feature set is pruned. Notably, the eleven-feature configuration in the ANN model exemplifies a consistent high-risk prediction AUC of 0.87, with significant p-values indicating robustness against feature diminution.

	Number of features	AUC high	AUC intermediate	AUC low	p-value
ANN	12 features	0.87 (0.83, 0.91)	0.61 (0.56, 0.65)	0.64 (0.61, 0.67)	0.001364
	11 features	0.87 (0.82, 0.91)	0.66 (0.62, 0.7)	0.83 (0.79, 0.87)	0.006442
	10 features	0.78 (0.74, 0.82)	0.58 (0.55, 0.61)	0.78 (0.74, 0.81)	0.014848
	9 features	0.76 (0.72, 0.81)	0.65 (0.61, 0.7)	0.83 (0.79, 0.87)	0.000943
	8 features	0.79 (0.75, 0.84)	0.61 (0.57, 0.65)	0.79 (0.75, 0.82)	0.003039
	7 features	0.82 (0.78, 0.86)	0.68 (0.63, 0.73)	0.82 (0.78, 0.85)	0.014543
	6 features	0.72 (0.68, 0.76)	0.65 (0.61, 0.69)	0.69 (0.64, 0.73)	0.313042
XGBoost	12 features	0.71 (0.66, 0.77)	0.52 (0.45, 0.58)	0.65 (0.60, 0.71)	4.13E−13
	11 features	0.73 (0.68, 0.79)	0.54 (0.49, 0.60)	0.65 (0.60, 0.70)	3.24E−10
	10 features	0.75 (0.70, 0.81)	0.51 (0.45, 0.58)	0.65 (0.60, 0.70)	1.01E−11
	9 features	0.74 (0.68, 0.79)	0.51 (0.45, 0.58)	0.65 (0.60, 0.71)	7.35E−12
	8 features	0.8 (0.75, 0.86)	0.56 (0.50, 0.61)	0.69 (0.64, 0.74)	9.21E−10
	7 features	0.80 (0.74, 0.85)	0.57 (0.52, 0.63)	0.70 (0.65, 0.76)	1.45E−11
	6 features	0.80 (0.75, 0.86)	0.57 (0.51, 0.62)	0.67 (0.61, 0.72)	1.47E−10
RF	12 features	0.79 (0.74, 0.85)	0.49 (0.49, 0.49)	0.77 (0.71, 0.84)	7.20E−18
	11 features	0.79 (0.74, 0.85)	0.49 (0.42, 0.55)	0.78 (0.71, 0.84)	4.28E−17
	10 features	0.79 (0.74, 0.85)	0.48 (0.42, 0.55)	0.78 (0.78, 0.78)	9.39E−18
	9 features	0.80 (0.75, 0.86)	0.50 (0.43, 0.55)	0.77 (0.72, 0.83)	7.06E−16
	8 features	0.81 (0.63, 0.99)	0.54 (0.54, 0.54)	0.76 (0.76, 0.76)	7.10E−184
	7 features	0.82 (0.77, 0.88)	0.54 (0.54, 0.54)	0.78 (0.78, 0.78)	7.85E−17
	6 features	0.83 (0.64, 1)	0.56 (0.56–0.56)	0.74 (0.74, 0.74)	1.20E−183
SVM	12 features	0.62 (0.62–0.62)	0.57 (0.5, 0.63)	0.75 (0.75, 0.75)	5.66E−19
	11 features	0.62 (0.62, 0.62)	0.59 (0.53, 0.65)	0.75 (0.69, 0.80)	5.06066E−13
	10 features	0.61 (0.55, 0.67)	0.58 (0.52, 0.65)	0.75 (0.69, 0.80)	1.20E−12
	9 features	0.691 (0.55, 0.68)	0.59 (0.54, 0.65)	0.74 (0.68, 0.80)	4.77E−10
	8 features	0.67 (0.61, 0.74)	0.75 (0.75, 0.75)	0.55 (0.50, 0.61)	9.21E−12
	7 features	0.75 (0.75, 0.75)	0.73 (0.67, 0.79)	0.45 (0.39, 0.52)	1.59E−17
	6 features	0.71 (0.71, 0.71)	0.67 (0.67, 0.67)	0.56 (0.51, 0.62)	3.05E−14
KNN	12 features	0.81 (0.63, 0.99)	0.56 (0.56, 0.56)	0.69 (0.69, 0.69)	3.80E−183
	11 features	0.81 (0.75, 0.88)	0.56 (0.56, 0.56)	0.69 (0.69, 0.69)	1.63E−20
	10 features	0.81 (0.63, 0.99)	0.56 (0.56, 0.56)	0.69 (0.69, 0.69)	3.80E−183
	9 features	0.81 (0.75, 0.88)	0.56 (0.56, 0.56)	0.69 (0.69, 0.69)	1.63E−20
	8 features	0.81 (0.63, 0.99)	0.56 (0.56, 0.56)	0.69 (0.69, 0.69)	3.80E−183
	7 features	0.81 (0.63, 0.99)	0.56 (0.56, 0.56)	0.69 (0.69, 0.69)	3.80E−183
	6 features	0.82 (0.76, 0.89)	0.56 (0.56, 0.56)	0.69 (0.69, 0.69)	1.0622E−19
RBF	12 features	0.59 (0.55, 0.64)	0.69 (0.64, 0.74)	0.73 (0.68, 0.78)	2.03E−03
	11 features	0.52 (0.48, 0.55)	0.63 (0.59, 0.66)	0.69 (0.66, 0.74)	3.52E−02
	10 features	0.63 (0.60, 0.67)	0.68 (0.62, 0.73)	0.68 (0.63, 0.72)	7.25E−01
	9 features	0.83 (0.80, 0.87)	0.68 (0.68, 0.68)	0.64 (0.59, 0.69)	1.67E−03
	8 features	0.73 (0.69, 0.76)	0.62 (0.57, 0.66)	0.68 (0.64, 0.73)	3.65E−01
	7 features	0.75 (0.72, 0.79)	0.67 (0.62, 0.72)	0.64 (0.60, 0.68)	1.77E−01
	6 features	0.89 (0.83, 0.93)	0.64 (0.61, 0.68)	0.74 (0.69, 0.79)	1.22E−04

The narrative shifts when considering the XGBoost model, where a decrease in AUC for intermediate risk prediction was noted with feature reduction, with the most substantial performance, an AUC of 0.71 (p = 4.13E−13) (Table 3), observed with the full set of 12 features. This indicates that XGBoost might rely on the complex interactions of multiple features to maintain predictive accuracy.

Similarly, the RF model exhibited a preference for eleven features, excelling particularly in low-risk prediction with an AUC of 0.76 (p = 7.10E−184) (Table 3), reinforcing the notion that an optimally proximate feature set can be identified that balances performance with model simplicity.

Conversely, SVM and KNN models demonstrated a more complex relationship between feature quantity and prediction accuracy, with the data not delineating an optimal feature set, hinting at more nuanced interactions between feature selection and model output.

In an interesting development, the performance of the RBF network in predicting high risk was bolstered by reducing the feature count to six, yielding an AUC of 0.89 (p = 1.22E−04) (Table 3). This finding underscores the potential of a highly influential and concentrated feature set to significantly enhance model performance.

## Discussion

This study focuses on substructural biomarkers and utilizes the value of Shapley Additive exPlanations (SHAP) to determine the influence of each biomarker in predicting drug-related risks like Torsades de Pointes (TdP). By implementing feature importance mechanisms, this research method methodically assesses how each biomarker contributes to the overall predictive model. This approach not only enhances the accuracy of toxicity prediction but also elucidates the complex role of certain electrophysiological features and enriches our understanding of the biological mechanisms behind drug-induced effects.

In the context of this research, ANN, XGBoost, RF, SVM, KNN, and RBF are used to identify in-silico biomarkers that significantly impact drug toxicity evaluation. Twelve biomarkers were selected as input features, encompassing morphological aspects of the Action Potential (AP) like $\frac{dVm}{dt}_{repol}$, $\frac{dVm}{dt}_{max}$, $V{m}_{resting}$, $AP{D}_{90}$, $AP{D}_{50}$, and $AP{D}_{tri}$, calcium transient morphology like $Ca{D}_{90}$, $Ca{D}_{50}$, $C{a}_{tri}$, $C{a}_{Diastole}$, and features related to charge movement, namely qNet, and qInward.

By implementing the GS method, we determined the most optimal model and then used the SHAP approach to identify biomarkers significantly contributing to drug risk classification. The SHAP approach is based on coalition game theory, where SHAP values are calculated to describe the contribution of each attribute. Unlike heuristic approaches used in previous studies, the SHAP approach considers the prediction probabilities of all inputs in a machine learning context. Its dual nature allows both positive and negative SHAP values to have equal influence in making predictions. As a result, we calculated the average absolute SHAP scores to identify the biomarkers with the most significant contributions.

The feature importance levels from the SHAP results of the ANN model in Fig. 5a, highlight qInward, $AP{D}_{50}$, and $AP{D}_{90}$ as significant influences in the TdP risk assessment, aligning with Yoo et al. findings that emphasize the complexity of action potentials and calcium transients in proarrhythmic potential^15^. This conformity strengthens the validation of our model and illustrates its capacity to capture crucial electrophysiological dynamics critical for accurate drug toxicity prediction. Specifically, the prominence of qInward in the SHAP analysis reflects the intricate role of inward ion currents, especially calcium and sodium, in initiating and maintaining arrhythmogenic activity. This emphasizes the charge movement in drug-induced TdP risk discussed in Dutta et al. and Li et al.^10,11^. Furthermore, the sufficient importance of duration markers like $AP{D}_{50}$ and $AP{D}_{90}$ aligns with traditional electrophysiological theory and supports the CiPA initiative’s shift towards complex in silico assessment strategies.

This analysis highlights the interaction between various electrophysiological parameters, indicating a more complex relationship than previously understood in the context of TdP risk, where the focus was often solely on AP prolongation or ionic current changes. Our ANN model, enriched with a comprehensive set of biomarkers, not only strengthens existing electrophysiological insights but also reveals the inherent complexity of proarrhythmic potential in drug-induced cardiotoxicity. A deeper understanding from the SHAP feature importance analysis confirms the robustness of our ANN approach in navigating the complex landscape of TdP risk assessment. ANN tends to capture the non-linear and complex interactions between features, using a deep architecture with multiple layers and neurons to model these relationships in detail, allowing it to detect very specific nuances and patterns in the data.

The SHAP feature importance results for our XGBoost model, shown in Fig. 5b, provide nuanced insights into the critical electrophysiological determinants for TdP risk assessment. The prominence of qInward and $\frac{dVm}{dt}_{repol}$ confirms the importance of inward ion flux and repolarization dynamics in modulating cardiac risk, in line with findings from Yoo et al. and Dutta et al.^11,15^, where a complex balance of ion currents underlies proarrhythmic potential. The model’s emphasis on $AP{D}_{90}$ along with $\frac{dVm}{dt}_{Max}$ indicates comprehensive electrophysiological interactions affecting arrhythmogenicity, highlighting the need for a holistic approach in cardiac safety evaluation, as also suggested in the CiPA comprehensive in silico modeling framework.

In the XGBoost model, the prioritization of features like qNet underscores the critical role of Channel block handling in cardiac toxicity, in line with the nuanced insights provided by Li et al.^55^, where qNet is an integral part of TdP risk prediction. This alignment underlines the relevance of these biomarkers in reflecting the multifactorial nature of drug-induced arrhythmia. Moreover, the presence of $V{m}_{Resting}$ in the analysis, albeit with a lower importance score, indicates the subtle impact of resting membrane potential on cardiac electrophysiology, necessitating further investigation as discussed in broader cardiac safety research.

Our analysis through SHAP feature importance levels for the XGBoost model not only aligns with established electrophysiological paradigms but also offers detailed exploration into how these biomarkers interact within the predictive model, enhancing current understanding of TdP risk. Integrating comprehensive biomarker analysis within the machine learning framework represents an advance in refining drug safety assessment, leveraging the strength of XGBoost to dissect complex biomarker interactions in predicting proarrhythmic risk. Although our findings illuminate the way forward in using sophisticated analytical models to capture the complex landscape of cardiac arrhythmogenicity, features with greater influence in predicting the target are given higher priority, indicating that XGBoost is effective in feature selection and handling nonlinear data, using gradient boosting techniques to enhance prediction.

On the other hand, the SHAP feature importance results for the RF model (Fig. 5c) identify qInward and qNet as the most influential factors in determining TdP risk, aligning with the critical role these features play in drug-induced arrhythmogenicity as discussed in studies by Yoo et al., and Li et al.^10,15,55^. The importance of qInward in the model underscores the fundamental role of inward ion currents, particularly through calcium and sodium channels, in shaping the cardiac action potential and inducing arrhythmias. This is consistent with the comprehensive analysis conducted in the CiPA initiative, which emphasizes the multifactorial nature of TdP risk, beyond the traditional focus on hERG channel blocking.

Additionally, the significance of $AP{D}_{90}$ and $C{a}_{Diastole}$ in the RF model highlights the complex interaction between action potential duration and calcium homeostasis in cardiac electrophysiology, reflecting the intricate insights provided by Dutta et al. about regulated ion channel activity affecting cardiac repolarization^11^. This analysis reveals a sophisticated landscape where subtle variations in these parameters can significantly alter the proarrhythmic potential of drugs, reinforcing the need to integrate a broad spectrum of biomarkers for more accurate and holistic risk assessment.

It can be concluded that the RF model, employing an ensemble approach, combines predictions from many decision trees to improve accuracy and reliability. RF tends to give equal weight to features and is good at identifying feature importance through its process.

The feature importance results from the SVM model, shown in Fig. 5d, indicate equal priority of biomarkers for assessing TdP risk. The importance of $Ca{D}_{50}$ and qInward in this analysis reaffirms the critical role of calcium dynamics and inward ion flow, respectively, in mediating arrhythmic potential, a finding that reflects the physiological basis emphasized in the CiPA initiative. Notably, the SVM model’s emphasis on $AP{D}_{90}$ and qNet aligns with the electrophysiological criteria discussed by Dutta et al. and Li et al.^11,55^, where these parameters are vital in representing the proarrhythmic tendencies of drugs through detailed action potential profiles and ion charge characteristics.

The lower ranking of features like $\frac{dVm}{dt}_{max}$ and $AP{D}_{50}$ in importance challenges the conventional consideration of their roles, indicating that TdP risk associated with drug compounds may not directly correspond with typical markers of cellular activity or repolarization duration. This insight challenges traditional views and signifies more complex interactions in arrhythmogenesis, requiring a broader perspective in risk stratification.

Furthermore, the SVM analysis reflects a sophisticated understanding of how various biomarkers collectively inform proarrhythmic risk, advocating a multifaceted approach in cardiac safety evaluation as supported by the advanced ANN methodology of Yoo et al.^15^. The depth of this analysis enhances the precision of TdP risk assessment, providing a compelling argument for the integration of diverse electrophysiological features beyond standard APD measurements.

At its core, the SHAP feature importance results for the SVM model significantly contribute to the evolving narrative of cardiac safety pharmacology, affirming the intricate balance of ionic and electrophysiological parameters in mediating drug-induced TdP risk. Meanwhile, the SVM model operates by seeking an optimal hyperplane to separate classes in feature space, with a strong focus on features that help define the decision margin. The use of kernels, such as RBF, allows SVM to work effectively in high-dimensional spaces.

The SHAP feature importance analysis for our KNN model, represented in Fig. 5e, identifies $Ca{D}_{90}$, $AP{D}_{90}$, $AP{D}_{50}$, $Ca{D}_{50}$, and other electrophysiological parameters as critical in assessing the drug-induced TdP risk. The importance of calcium transient durations ($Ca{D}_{90}$ and $Ca{D}_{50}$) along with action potential durations ($AP{D}_{90}$ and $AP{D}_{50}$) aligns with findings from Dutta et al. and Yoo et al.^11,15^, underscoring the significance of these parameters in cardiac toxicity prediction. This alignment reaffirms the relevance of temporal dynamics of calcium handling and action potential in mediating arrhythmic vulnerability, as proposed in the CiPA initiative. The emphasis on these biomarkers by the KNN model reflects the multifactorial nature of TdP risk, highlighting not only ionic current changes but also the broader electrophysiological landscape affecting arrhythmogenic potential.

Furthermore, the SHAP analysis elucidates the contributions of less emphasized features like $\frac{dVm}{dt}_{max}$, qNet, and $\frac{dVm}{dt}_{repol}$, suggesting a more complex and integrative approach to understanding proarrhythmic risk beyond conventional single-parameter assessment. This perspective aligns with the tiered complexity observed in real-world cardiac electrophysiology, where multiple interdependent factors converge to influence arrhythmia risk. By capturing the multifaceted interactions of electrophysiological biomarkers, the KNN model offers a sophisticated lens through which the subtle intricacies of drug-induced arrhythmia threats can be recognized, aligning with the broader goal of enhancing prediction accuracy and reliability in pre-clinical cardiac safety assessment.

This comprehensive analysis not only reinforces the critical electrophysiological foundation identified in previous research but also advances our understanding by showcasing the KNN model’s capacity to integrate and prioritize a wide spectrum of biomarkers. The KNN model makes predictions based on the proximity of sample data to its k nearest neighbors, with features influencing the measurement of proximity or distance. This model is highly intuitive and non-parametric, relying on the local data structure.

Figure 5f illustrates the feature importance levels of the RBF model, showcasing the ranking of features based on their impact on the model’s output. In this analysis, $\frac{dVm}{dt}_{max}$ and $\frac{dVm}{dt}_{repol}$ emerge as the top two features, indicating their significant influence on the model’s predictions, followed by features like $C{a}_{tri}$, $AP{D}_{tri}$, $AP{D}_{90}$, and others.

In the context of drug-induced proarrhythmic evaluation, as discussed in the study by Yoo et al.^15^, these features (especially $\frac{dVm}{dt}_{max}$ and $AP{D}_{90}$) are critical in assessing the action potential morphology and calcium transients of cardiomyocytes, which are crucial for understanding drug-induced arrhythmias. The emphasis on $\frac{dVm}{dt}_{max}$ (the maximum rate of voltage change during depolarization) and $AP{D}_{90}$ (the duration of action potential at 90% repolarization) aligns with their physiological importance in cardiac electrophysiology and their recurrence as significant predictors in arrhythmogenic risk assessment.

The significance of $\frac{dVm}{dt}_{max}$ and $AP{D}_{90}$ in this study can be linked to their crucial roles in the formation and propagation of cardiac action potentials, and how changes in these parameters are indicative of proarrhythmic risk. This analysis supports the utility of machine learning models in identifying key physiological indicators of drug-induced arrhythmias and enhances understanding of the underlying mechanisms of these adverse effects. Moreover, the presence of calcium-related features like $Ca{D}_{50}$ and $Ca{D}_{90}$ in the importance ranking further reinforces the significance of calcium dynamics in cardiac function and its disruption in drug-induced proarrhythmia.

Overall, this study reveals that the qInward parameter significantly influences all levels of drug risk prediction—high, intermediate, or low—when using ANN, XGBoost, RF, and SVM models. This observation aligns with the research conducted by Li et al.^10^, where qInward was identified as an effective biomarker for differentiating levels of drug-induced TdP risk. However, it’s important to note that findings from the Food and Drug Administration (FDA) suggest that qNet is a more crucial feature in distinguishing drug risk levels, specifically when using a dynamic hERG model^55^.

Contrary to previous studies, we utilized a conventional hERG model without dynamic parameter modifications. This decision was made with the primary goal of maintaining the integrity of experimental data before involving MCMC methods. We committed to preserving experimental conditions prior to the MCMC process so that the results could be more directly compared with existing empirical data. Through this approach, we aim to provide a clearer and more measurable contribution to potential biomarkers, in classifying drug risk, without affecting the foundational experimental conditions of this study.

On the other hand, in other classification models like SVM, KNN, and RBF, different features emerged as important. In the SVM and KNN models, biomarkers indicating the duration of calcium transients (such as $Ca{D}_{50}$, $Ca{D}_{90}$, and $C{a}_{tri}$) play a crucial role in classifying drug risk levels, though the SVM model still retains qInward as an important feature in predicting drug risk levels. However, in the KNN model, features indicating the duration of action potential and calcium transients are key in predicting the TdP drug risk level. This contrasts with the RBF model, where the most critical features are $\frac{dVm}{dt}_{max}$ and $\frac{dVm}{dt}_{repol}$, highlighting the diversity in feature importance across different models and the complexity of accurately predicting drug-induced TdP risk.

To understand how the SHAP (SHapley Additive exPlanations) waterfall plot depicts the contribution of each feature to the model’s prediction at different risk levels (high, intermediate, and low), we need to examine the specific characteristics of each feature within the context of various machine learning models (ANN, XGBoost, RF, SVM, KNN, and RBF). These plots are crucial for understanding how different features influence drug toxicity risk predictions in each model.

In the context of high-risk prediction using the ANN model, features like $AP{D}_{50}$ and $AP{D}_{90}$ gain significant weight. $AP{D}_{50}$ refers to the action potential duration at which 50% of repolarization is achieved, while $AP{D}_{90}$ indicates the time to reach 90% repolarization. The emphasis on these features suggests that the ANN model considers repolarization duration as a critical indicator of high toxicity risk. This importance is based on the insight that these parameters provide information on how a drug affects the cardiac cell’s ability to recover after depolarization, with longer durations indicating a higher potential for arrhythmia risk.

For intermediate risk scenarios, the ANN model shifts its focus to dynamic features like $\frac{dVm}{dt}_{max}$, representing the maximum rate of voltage change during repolarization, and calcium-related features like $C{a}_{tri}$, which may relate to intracellular calcium concentration. The importance of these features indicates that changes in voltage dynamics and calcium handling become more pivotal in assessing intermediate-level toxicity risk. This change signifies that ANN prioritizes dynamic and responsive cell aspects under intermediate risk conditions, reflecting a more dynamic and adaptive understanding of the drug’s interaction with the cardiovascular system.

In predicting low risk, the ANN model places greater emphasis on calcium dynamics, particularly through features like $Ca{D}_{50}$ and $C{a}_{Diastole}$. $Ca{D}_{50}$ relates to the duration over which 50% of calcium is repolarized, while $C{a}_{Diastole}$ refers to the calcium level during the diastolic phase. Focusing on these features for low-risk assessment suggests that the ANN model regards changes in calcium homeostasis as a critical factor in determining lower toxicity potential. The model’s adaptation to the more subtle electrophysiological nuances in low-risk conditions highlights its sensitivity to minor changes in calcium parameters, which may indicate a greater safety margin against toxic effects.

In high-risk classification, XGBoost places strong emphasis on features like qInward and qNet, indicating that the model is highly sensitive to changes in inward ionic currents and total net current, which can be key indicators of high toxicity. The qInward feature, potentially representing the inward current components during the action potential cycle, becomes central in assessing high-risk potential. This focus suggests that XGBoost associates significant changes in this parameter with increased toxicity potential.

XGBoost focuses on calcium dynamics for intermediate-risk predictions, with features like $Ca{D}_{90}$ and $C{a}_{tri}$ becoming more prominent. $Ca{D}_{90}$, referring to the duration at 90% calcium repolarization, and $C{a}_{tri}$, possibly related to intracellular calcium levels, are crucial in determining intermediate-risk toxicity. This approach indicates that XGBoost is sensitive to how changes in cellular calcium handling can reflect a shift in risk level from high to intermediate, emphasizing the importance of calcium signaling in toxicity assessment.

The model highlights the importance of action potential duration and calcium dynamics in low-risk classification, mainly through features like $AP{D}_{90}$ and $C{a}_{Diastole}$. APD_90 represents the time required for 90% of the action potential to repolarize, while $C{a}_{Diastole}$ relates to diastolic calcium concentration. These features become more relevant in identifying low toxicity risk, suggesting that XGBoost considers subtle changes in electrophysiological and calcium dynamics as significant indicators of lower toxicity.

RF model adopts a balanced approach in assessing high risk, giving significant weight to features like qInward and $AP{D}_{90}$. qInward, related to inward ionic currents, and $AP{D}_{90}$, measuring 90% of the action potential duration, are crucial in identifying high-risk situations. This indicates that RF recognizes both aspects—ionic currents and action potential duration—as key indicators that can reflect the high toxicity potential of drugs. This approach demonstrates the model’s comprehensive understanding of the factors contributing to high risk, integrating diverse electrophysiological inputs for accurate assessment.

In the context of intermediate risk, RF focuses on features associated with repolarization dynamics and calcium, such as $\frac{dVm}{dt}_{repol}$ and $Ca{D}_{50}$. The feature $\frac{dVm}{dt}_{repol}$, measuring the rate of voltage change during repolarization, and $Ca{D}_{50}$, potentially indicating calcium concentration at 50% of the diastolic phase, become more pronounced. This signifies that the model prioritizes understanding how cells return to electrical stability and how calcium is regulated in intermediate-risk conditions, which is crucial for assessing moderate toxicity levels.

RF exhibits a holistic approach to low-risk predictions by emphasizing various electrophysiological signals. Features covering different aspects of cellular function, such as qInward, $Ca{D}_{50}$, $AP{D}_{90}$, and others, all contribute to assessing low toxicity risk. This approach shows that RF considers various aspects of electrophysiological data to form a comprehensive view of low-risk potential, integrating information from multiple sources to make balanced and informed predictions.

SVM model emphasizes explicitly features such as qInward and $C{a}_{Diastole}$ when assessing high risk. This indicates that SVM regards changes in inward ionic current (qInward) and diastolic calcium levels ($C{a}_{Diastole}$) as essential indicators of high-risk potential. By prioritizing these features, SVM highlights their correlation with significant toxicity events, reflecting its sensitivity to ionic and calcium dynamics changes that can lead to severe cardiotoxic effects.

SVM adjusts its focus for intermediate-risk predictions to highlight elements like $Ca{D}_{50}$ and qNet. $Ca{D}_{50}$, related to 50% calcium repolarization duration, becomes significant in the context of intermediate risk, indicating that SVM considers how calcium repolarization duration contributes to moderate toxicity levels. Moreover, qNet, representing the total net current, emerges as another key feature, showing that SVM acknowledges the importance of integrating ionic current information in toxicity risk assessment.

In the low-risk category, SVM adopts a subtler approach, focusing on features associated with action potential dynamics. In contrast, within the KNN algorithm, the primary focus on high risk is evident in features like $Ca{D}_{90}$ and $AP{D}_{90}$. $Ca{D}_{90}$, measuring the duration of 90% calcium repolarization, and $AP{D}_{90}$, indicating the duration until 90% of the action potential repolarization, becomes significant. The emphasis on these features reflects KNN’s recognition of the importance of prolonged electrophysiological events in signaling high toxicity risk. This shows that KNN considers extended durations in calcium activity and action potential as key high risk indicators, reflecting its sensitivity to potentially dangerous cardiovascular dynamics.

KNN shows a balanced assessment of features such as $\frac{dVm}{dt}_{max}$ and qInward for intermediate risk. $\frac{dVm}{dt}_{max}$, representing the maximum rate of voltage change during repolarization, and qInward, indicating the inward current, both receive attention, highlighting how KNN evaluates various aspects of electrophysiological dynamics to classify intermediate risk. This shows the model’s mature understanding of the complex interactions between various electrophysiological factors in determining toxicity risk.

At low risk, KNN adopts a comprehensive approach, considering various electrophysiological features. This shows that KNN does not solely focus on one or two features in assessing low risk but integrates various signals to form a holistic view of toxicity potential. This approach allows KNN to effectively differentiate between different risk profiles, leveraging a broad understanding of electrophysiological dynamics that contribute to low toxicity occurrences. It illustrates KNN’s sensitivity to electrophysiological nuances and calcium dynamics that characterize low-risk situations, using this information to distinguish between different risk levels accurately.

For the RBF model, biomarkers like $\frac{dVm}{dt}_{repol}$ and $AP{D}_{90}$ are particularly prominent when analyzing high-risk drugs. The feature $\frac{dVm}{dt}_{repol}$, which measures the rate of membrane potential change during repolarization, along with $AP{D}_{90}$, the duration until 90% of the action potential repolarization, becomes critical. This indicates that RBF focuses explicitly on the dynamics of repolarization and the duration of action potential in the context of high risk, associating changes in these parameters with increased toxicity potential. The model considers significant changes in these features as key indicators of heightened toxicity, emphasizing the importance of understanding these electrophysiological processes in risk prediction.

RBF emphasizes the importance of features like $\frac{dVm}{dt}_{repol}$ and qNet for intermediate risk. This reflects the model’s adaptation to more moderate risk predictions, where the dynamics of repolarization $\frac{dVm}{dt}_{repol}$) and the total net current (qNet) plays a crucial role. This approach underscores RBF’s understanding of how integrating repolarization dynamics and ionic current information can assist in differentiating between high and intermediate risk, highlighting the significance of these features in characterizing toxicity levels.

RBF employs a versatile approach in classifying low risk, emphasizing a broad spectrum of features from $\frac{dVm}{dt}_{repol}$ to $C{a}_{Diastole}$. The feature $C{a}_{Diastole}$, associated with the calcium level at the end of the diastolic phase and other parameters, becomes relevant in identifying low risk. This reflects RBF’s ability to interpret various electrophysiological signals, using a diverse feature set to predict low toxicity risk accurately.

In this study, several machine learning models were used, incorporating 12 in-silico biomarkers as input features. Using ANN, XGBoost, RF, SVM, KNN, and RBF, the training process was conducted with 12 drug datasets, each containing 12 in-silico biomarkers as input features $\frac{dVm}{dt}_{repol}$, $\frac{dVm}{dt}_{max}$, $V{m}_{resting}$, $AP{D}_{90}$, $AP{D}_{50}$, $AP{D}_{tri}$, $Ca{D}_{90}$, $Ca{D}_{50}$, $C{a}_{tri}$, $C{a}_{Diastole}$, qInward, and qNet). The training process utilized fivefold cross-validation, retaining the model with the smallest validation loss for testing. Following the Comprehensive in vitro Proarrhythmia Assay (CiPA) criteria, testing involved 10,000 iterations, each comprising 16 drug datasets showing different drug sample combinations^15,29,55^. Each drug combination test determined the classification system’s performance for each model. Subsequently, considering the results from the feature importance plot, biomarkers were eliminated one by one according to their overall importance level.

The ANN model showed the highest Area Under Curve (AUC) classification performance when $V{m}_{resting}$ was removed, achieving AUC of 0.92 (0.88–0.96) for high-risk levels, 0.83 (0.73–0.9) for intermediate-risk levels, and 0.98 (0.95–0.98) for low-risk levels (Table 2). However, it should be noted that there was variability in AUC for predicting intermediate-risk levels due to similarities between high and intermediate risk groups. The analysis of critical features also revealed this differentiation, with the feature contribution to high-risk prediction being lower than for intermediate risk. In the ANN model, removing $Ca{D}_{90}$ resulted in a performance decrease to 0.79, indicating that optimal accuracy was achieved using only eleven biomarkers.

Meanwhile, other classification models like Random Forest (RF) and XGBoost are discussed in Table 2. Optimal system performance in the Random Forest model was achieved by eliminating six biomarkers: $C{a}_{tri}$, $AP{D}_{tri}$, $\frac{dVm}{dt}_{repol}$, $Ca{D}_{90}$, $Ca{D}_{50}$, and $\frac{dVm}{dt}_{max}$. The Random Forest classification model yielded the best AUC scores of 0.75 (0.5–0.96) for high risk and 0.71 (0.62–0.81) for low risk (Table 2). Similarly, the XGBoost model showed comparable AUC scores of 0.71 (0.5–0.96) for high risk and 0.71 (0.62–0.81) for low risk (Table 2). Despite removing the six lowest-ranking biomarkers, their accuracy remained unchanged in the XGBoost classification model. Therefore, the overall best system performance in the XGBoost model was achieved by removing only one biomarker ($C{a}_{tri}$).

The Support Vector Machine (SVM) model with seven biomarkers ($Ca{D}_{50}$, qInward, $AP{D}_{90}$, qNet, $\frac{dVm}{dt}_{max}$, $Ca{D}_{90}$, $\frac{dVm}{dt}_{repol}$) showed varying performance across risk levels. At high risk, SVM achieved an AUC of 0.71, indicating good accuracy but less than that achieved by ANN. For intermediate risk, SVM showed a lower AUC than the ANN model, suggesting that SVM might struggle to differentiate between high and intermediate risk. This is further emphasized by the relatively low positive likelihood ratio (LR+) for high-risk samples. However, for low risk, SVM performed quite well, with an AUC nearly the same as that of ANN.

The k-Nearest Neighbors (KNN) model, after eliminating six biomarkers $\frac{dVm}{dt}_{max}$, qNet, $\frac{dVm}{dt}_{repol}$, $C{a}_{Diastole}$, $V{m}_{Resting}$, qInward), had a performance similar to SVM in terms of AUC for high and intermediate risk but appeared slightly better in predicting low risk. KNN is known to be sensitive to the selected features because its algorithm depends on the proximity of these features in feature space. This result suggests that feature reduction may have facilitated the model in identifying patterns associated with low risk in the data.

RBF model showed AUC values similar to SVM and KNN for high risk but stood out with better performance in predicting low risk, indicating that the RBF kernel function might be more effective in mapping features to a higher-dimensional space to distinguish low risk from other risks. Good positive and negative likelihood ratio (LR+ and LR−) results also indicate that the RBF model is quite reliable in classifying high and low risk.

These findings highlight the importance of feature selection and the impact of different classification models on the accuracy of drug risk level predictions. Reducing the number of biomarkers as input features in classification models does not always lead to improved classification capabilities. This is because the complexity of interactions among the features can sometimes be nonlinear or not directly apparent. In some cases, eliminating features that seem irrelevant or contribute minimally can reduce the model’s ability to capture essential patterns in the data. In other words, features that are individually considered less significant can contribute to complex interactions with other features, and their removal can diminish the information needed by the model to make accurate predictions. Therefore, in developing classification models, it is crucial to consider the presence of each feature and how these features interact with each other in a broader context.

Moreover, the system’s performance was evaluated based on diagnostic accuracy, as suggested by Li and Simundic^55,32^. The obtained AUC values for the high and low-risk groups are within the "outstanding" evaluation criteria, with AUCs of 0.92 and 0.98, respectively. However, the intermediate risk group falls within the "good" accuracy range for the ANN model, with an AUC of 0.83. Additionally, the positive likelihood ratio (LR+) values are greater than 5 for the high and low-risk groups in the ANN model, classified as "outstanding" according to Li’s criteria and also indicating good performance according to the criteria proposed by Šimundic^32^.

In a more detailed analysis of the diagnostic accuracy performance conducted by Li and Simundic^55,32^, it is evident that the XGBoost and RF classification models consistently meet the specified benchmarks, providing vital predictive accuracy for high and low-risk levels as evidenced by their Area Under the Curve (AUC), positive likelihood ratio (LR+), and negative likelihood ratio (LR−) values. The SVM model shows commendable AUC scores for high and low risk, yet its performance appears dimmer for intermediate risk, indicating potential limitations of SVM in differentiating between high and intermediate risk levels. This could be related to nuances in selecting hyperparameters or characteristics of the kernel used. SVM’s strength lies in its ability to transform the feature space through kernel functions, but this can also obscure which features are most influential, especially in datasets where features are complexly correlated or intricately intertwined.

KNN model, with its intuitive approach based on feature proximity, produces commendable Area Under the Curve (AUC) scores for high and low risk but shows a decrease in performance for intermediate risk. The effectiveness of this model depends on the correct selection of the number of neighbors and the distance metric, where suboptimal tuning can result in reduced sensitivity to the subtle gradations among different risk levels. Challenges for KNN may arise if the data distribution is uneven or imbalanced among the various risk categories.

RBF, often used in the SVM framework for its kernel function, shows consistent performance across all risk categories, particularly excelling in identifying low-risk cases. This suggests that the RBF model might adeptly navigate the complexities in the data, effectively separating low-risk cases. However, similar to SVM, the use of the RBF kernel can obscure the interpretability of the model and the significance of individual features.

While the performance of these models does not surpass that of the ANN classification model, our findings align with previous research by Yoo et al.^15^, who developed an ANN model integrating nine in-silico features. This model, encapsulating morphological insights from action potential traces, calcium transient traces, and net ionic charge features, achieved an AUC of 0.92 for high risk, 0.83 for intermediate risk, and 0.98 for low risk^15^. Although the authors suggest a slightly better system efficacy for the intermediate-risk AUC, their analysis did not delve into the contribution of in-silico features in drug risk level classification using XAI, highlighting an area for further exploration in the nuanced landscape of model-based diagnostics.

A more detailed analysis based on the provided table would integrate various AUC, accuracy, and likelihood ratio (LR) values for each model, shedding light on assessing drug risk levels. In the ANN model, removing $V{m}_{resting}$ improved AUC for high risk to 0.92, but performance for intermediate risk decreased when $Ca{D}_{90}$ was excluded. In contrast, in the XGBoost, RF, SVM, KNN, and RBF models, eliminating the six lowest-ranked biomarkers did not alter accuracy, indicating that these features might not provide significant contributions or that complex interactions remain uncovered.

Comparing these metrics with studies by Yoo et al. and Li et al.^15,55^, the ANN model is confirmed to have superior diagnostic performance, even with varying features. This evaluation reveals success in identifying high- and low-risk cases, but it also presents challenges in the intermediate-risk scenario. This emphasizes the crucial role of accurate feature selection and how modifying specific features can affect the overall performance of the classification model.

Optimal feature selection aims to enhance accuracy and delve into the contribution and interactions among features related to classification outcomes. Therefore, a meticulous evaluation of each feature is essential, considering their significance and their synergistic function within the model to produce the most accurate predictions.

Extensive testing involving 10,000 iterations through various classification models using drug datasets has revealed critical insights into the predictive performance and feature relevance of in-silico biomarkers. Specifically, this comprehensive analysis illuminates the impact of feature reduction on the model’s ability to classify different levels of drug risk accurately.

ANN model demonstrated optimal improvement in the Area Under the Curve (AUC), particularly for high-risk classification, using only 7 features. This significant reduction in the number of features, supported by relevant p-values, signifies success in filtering out less influential features, resulting in a leaner yet more accurate model. However, the performance showed variability for intermediate and low-risk classifications, suggesting that some eliminated features might contain important information for more accurate predictions at these risk levels.

For XGBoost, an increase in AUC for high risk was observed with feature reduction, indicating the effectiveness of feature selection in eliminating redundancy without sacrificing performance. Yet, performance declines for intermediate and low risks highlight the complexities in predictive modeling that require more detailed feature interactions.

RF model saw consistent AUC improvements for high risk with feature reduction, indicating the robustness of the RF model in mitigating overfitting and capturing essential patterns with a smaller feature set. However, for intermediate and low risks, a broader set of features may be necessary for accurate predictive modeling.

Conversely, SVM exhibited a consistent decrease in AUC with feature reduction, indicating this model’s need for a comprehensive feature set to maintain good predictive performance. This may reflect SVM’s sensitivity to high-dimensional feature space where the model can identify optimal separating hyperplanes.

KNN showed varied results; peak AUC was achieved with eleven features for high risk, while for low risk, the model remained robust even with fewer features. This indicates that KNN may require careful feature selection for high-risk but remains resilient for low-risk despite feature reduction.

Lastly, the RBF demonstrated increased AUC for high risk with fewer features, signifying its ability to capture complex patterns in the dataset. However, like other models, its performance decreases for intermediate and low risks when features are reduced.

Overall, these findings underscore the importance of careful and strategic feature selection in developing efficient classification models. Each model exhibits unique characteristics in response to feature reduction, with no one-size-fits-all rule applicable. Therefore, understanding individual contributions and synergistic interactions among features is crucial in designing robust and reliable classification systems for predicting drug risk in clinical practice.

This study has limitations in that the feature importance results from the ANN model are only sometimes clearly interpretable. In some cases, ANN needs to provide an intuitive explanation of feature contributions to prediction outcomes, making it challenging to derive deeper insights from the model and connect them with more robust scientific explanations. The higher complexity of the XGBoost model compared to methods like linear regression makes it a "black box" that is hard to interpret quickly. Despite techniques like feature importance and SHAP values, interpretation can still be complex, especially in highly intricate cases. The feature importance method used in Random Forest tends to be biased toward features with more categories or values, leading to inaccurate assessments of essential features with lower variation. These limitations arise because each method has its approach and characteristics in modeling data. Hence, these limitations underscore the importance of a comprehensive understanding of the data and its context when selecting and applying a particular classification method.

In conclusion, this study uses the ANN model to analyze the impact of optimal biomarker selection in predicting drug risk. qInward showed significant influence across all drug risk level predictions. At high risk levels, an extension of action potential duration ($AP{D}_{50}$ and $AP{D}_{90}$) was associated with decreased risk. Conversely, increased $AP{D}_{50}$ and $AP{D}_{90}$ at intermediate risk levels contributed positively. A higher calcium concentration at the diastolic stage ($C{a}_{Diastole}$) correlated with low and intermediate risk. $C{a}_{tri}$ negatively impacted the prediction of intermediate risk. The XGBoost model emphasized several biomarkers, particularly qInward, in risk prediction. Reducing less relevant input features or those with minimal contributions to the classification model can diminish the model’s ability to capture significant patterns. Feature selection in classification models is crucial, but reducing the number of input features only sometimes leads to improved model performance. Testing with various classification models and using fivefold cross-validation yielded different results, with the ANN model showing the highest AUC, emphasizing the $V{m}_{resting}$ feature. However, variability in accuracy occurred in intermediate-risk predictions due to similarities between high and intermediate-risk groups. Limitations in feature interpretation occur in complex ANNs, which tend to be biased towards features with more categories or values. All these limitations highlight the importance of a profound understanding of the data and context in selecting and implementing classification models.

## Supplementary Information

Supplementary Information.

## Data Availability

The original contributions generated for this study are fully included within the published article and its supplementary materials. The code used in this study is available on GitHub at https://github.com/kit-cml/XAI_optimal_biomarker , which includes detailed documentation, example datasets, and a guide for usage. For further inquiries or to access additional data related to this research, interested parties are encouraged to contact the corresponding author(s). We are committed to promoting transparency and accessibility in our research and will provide relevant data and materials upon reasonable request, ensuring compliance with our ethical and institutional guidelines.
