# Supplementary material for: Explainable artificial intelligence (XAI) to find optimal in-silico biomarkers for cardiac drug toxicity evaluation
Source: Sci Rep. 2024 Oct 14;14:24045. doi: 10.1038/s41598-024-71169-w (PMC11473646; doi:10.1038/s41598-024-71169-w)
Supplement: Supplementary file 1 — Supplementary Information. [file 41598_2024_71169_MOESM1_ESM.docx]

**SUPLEMENTARY FIGURES**


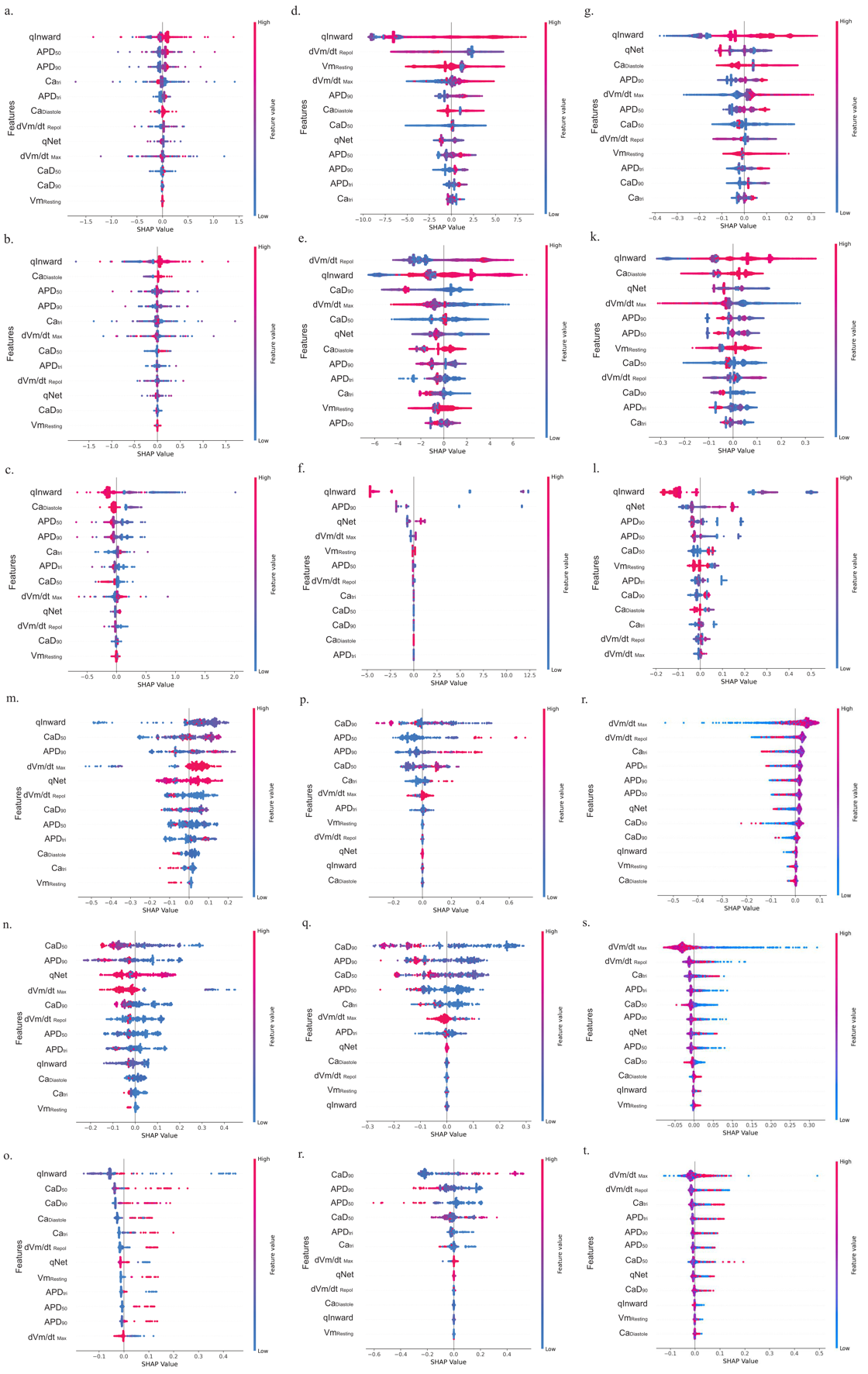


**Figure S1.** Comprehensive SHAP Value Analysis Across Machine Learning Models for Drug Risk Classification: This visualization presents the impact of electrophysiological feature contributions in drug toxicity predictions using SHAP values. Panels a-c depict the waterfall plots for the Artificial Neural Network (ANN), illustrating the feature importance for high, intermediate, and low-risk classifications, respectively. Panels d-f represent the XGBoost model's feature contribution, emphasizing the differences in electrophysiological dynamics critical for each risk level. For the Random Forest (RF) model, panels g-i show how diverse electrophysiological signals synthesize to assess drug risks. Panels j-l for the Support Vector Machine (SVM) model, m-o for the k-Nearest Neighbors (KNN), and p-r for the Radial Basis Function (RBF) model exhibit the varying significance of specific action potentials and calcium signaling phases, delineating the nuanced approach of each model in navigating the complex space of cellular behaviors for accurate risk classification.


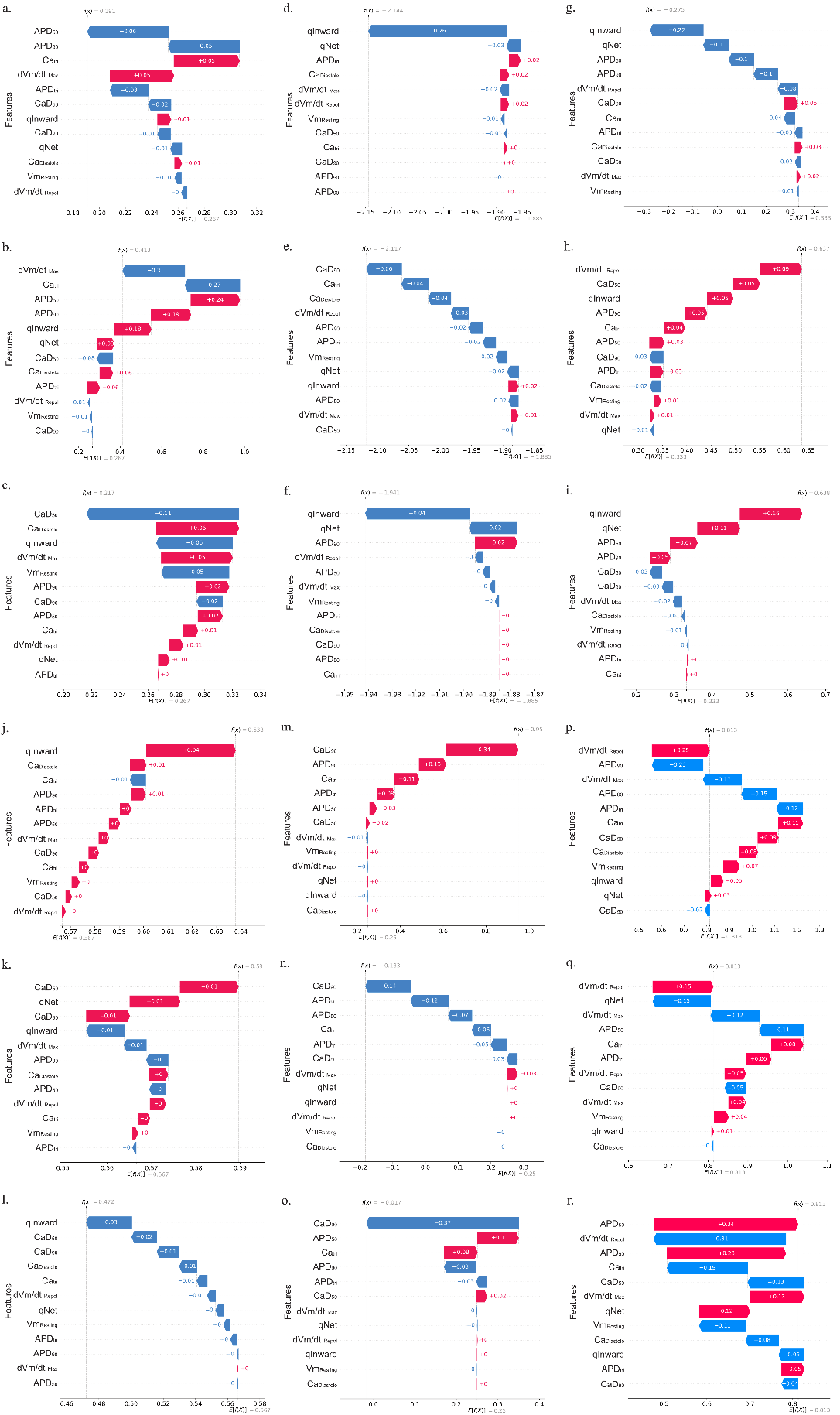


**Figure S2.** Investigating Electrophysiological Biomarkers in Drug Safety Assessment: SHAP Waterfall Plots for Insightful Model Interpretation. Panels a-c provide a visual explanation of feature importance in the ANN model, demonstrating the varying impact of features like APD50 and APD90 on high, intermediate, and low drug risk classifications. Panels d-f offer a glimpse into the XGBoost model's logic, highlighting the electrophysiological features critical for each class of drug risk. For the Random Forest (RF) approach, panels g-i aggregate diverse electrophysiological signals to evaluate drug risk comprehensively. SVM's analytical strategy is visualized in panels j-l, with the KNN model's methodology elucidated in panels m-o, focusing on key electrophysiological markers. Lastly, panels p-r reveal the Radial Basis Function (RBF) model's nuanced understanding of dynamic features that shape predictive patterns across different risk categories.

**SUPLEMENTARY TABLES**

Table S1. This table summarizes the performance results of an Artificial Neural Network (ANN) model tested 10,000 times with varying numbers of features, ranging from 6 to 12 features. The metrics used for evaluation include Accuracy (Acc), Area Under the Curve (AUC) for high, intermediate, and low prediction categories, and Positive Likelihood Ratio (LR+ ) for high, intermediate, and low prediction categories. For each feature configuration, the table presents the metric value and the 95% confidence interval (CI). These values indicate the model's performance with statistical details, providing insight into the variability and reliability of the ANN model's performance estimates under different testing conditions.

|  | **12 features** | **11 features** | **10 features** | **9 features** | **8 features** | **7 features** | **6 features** |
| --- | --- | --- | --- | --- | --- | --- | --- |
| **Acc** | 0.83 (0.75, 0.83) | 0.83 (0.79, 0.83) | 0.79 (0.75, 0.79) | 0.75 (0.71, 0.79) | 0.79 (0.71, 0.88) | 0.75 (0.67, 0.79) | 0.75 (0.67, 0.79) |
| **AUC High** | 0.96 (0.92, 1.0) | 0.92 (0.88, 0.96) | 0.94 (0.85, 1.0) | 0.85 (0.65, 0.96) | 0.83 (0.6, 0.92) | 0.92 (0.65, 1.0) | 0.77 (0.62, 0.98) |
| **AUC Intermediate** | 0.68 (0.59, 0.78) | 0.83 (0.73, 0.9) | 0.79 (0.67, 0.89) | 0.67 (0.54, 0.75) | 0.68 (0.59, 0.78) | 0.68 (0.56, 0.73) | 0.68 (0.6, 0.75) |
| **AUC Low** | 0.8 (0.76, 0.84) | 0.98 (0.95, 0.98) | 0.82 (0.78, 0.89) | 0.93 (0.84, 0.98) | 0.93 (0.82, 0.95) | 0.76 (0.65, 0.85) | 0.64 (0.55, 0.73) |
| **LR+ High** | 500001.0 (500000.06, 500001.99) | 500000.97 (250001.07, 500001.98) | 500000.94 (250000.79, 500001.96) | 250001.1 (1.0, 500001.72) | 250001.2 (3.0, 500001.77) | 4.0 (2.0, 9.0) | 3.0 (1.5, 9.0) |
| **LR+ Inter** | 2.25 (1.61, 2.25) | 2.25 (1.8, 2.25) | 1.8 (1.5, 1.8) | 1.5 (1.29, 1.8) | 2.57 (1.29, 3.86) | 2.57 (1.29, 3.21) | 2.57 (1.29, 3.21) |
| **LR+ Low** | 600000.69 (4.4, 600002.28) | 600000.92 (400000.53, 600002.16) | 400001.0 (400000.07, 400001.78) | 400000.82 (4.4, 400001.74) | 5.5 (2.93, 11.0) | 4.4 (2.2, 600001.31) | 4.4 (2.2, 400001.56) |
| **LR- High** | 0.5 (0.5, 0.5) | 0.5 (0.5, 0.75) | 0.5 (0.5, 0.75) | 0.75 (0.5, 1.0) | 0.55 (0.5, 0.82) | 0.3 (0.0, 0.67) | 0.33 (0.0, 0.75) |
| **LR- Inter** | 0.0 (0.0, 0.32) | 0.0 (0.0, 0.0) | 0.0 (0.0, 0.0) | 0.0 (0.0, 0.0) | 0.43 (0.18, 0.77) | 0.55 (0.37, 0.86) | 0.55 (0.26, 0.77) |
| **LR- Low** | 0.4 (0.4, 0.6) | 0.4 (0.4, 0.6) | 0.6 (0.6, 0.6) | 0.6 (0.6, 0.8) | 0.0 (0.0, 0.28) | 0.6 (0.4, 0.88) | 0.66 (0.6, 0.88) |

Table S2. This table summarizes the performance results of an XGBoost model tested 10,000 times with varying numbers of features, ranging from 6 to 12 features. The metrics used for evaluation include Accuracy (Acc), Area Under the Curve (AUC) for high, intermediate, and low prediction categories, and Positive Likelihood Ratio (LR+ ) for high, intermediate, and low prediction categories. For each feature configuration, the table presents the metric value and the 95% confidence interval (CI). These values indicate the model's performance with statistical details, providing insight into the variability and reliability of the XGBoost model's performance estimates under different testing conditions.

|  | **12 features** | **11 features** | **10 features** | **9 features** | **8 features** | **7 features** | **6 features** |
| --- | --- | --- | --- | --- | --- | --- | --- |
| **Acc** | 0.67 (0.58, 0.75) | 0.67 (0.58, 0.75) | 0.67 (0.58, 0.75) | 0.71 (0.58, 0.79) | 0.67 (0.58, 0.75) | 0.67 (0.58, 0.75) | 0.67 (0.58, 0.79) |
| **AUC High** | 0.73 (0.54, 0.9) | 0.75 (0.56, 0.9) | 0.77 (0.56, 0.92) | 0.79 (0.58, 0.92) | 0.81 (0.58, 0.94) | 0.81 (0.58, 0.94) | 0.81 (0.54, 0.94) |
| **AUC Intermediate** | 0.51 (0.38, 0.63) | 0.56 (0.43, 0.67) | 0.52 (0.4, 0.65) | 0.56 (0.43, 0.67) | 0.56 (0.43, 0.67) | 0.56 (0.41, 0.68) | 0.59 (0.46, 0.7) |
| **AUC Low** | 0.64 (0.55, 0.73) | 0.62 (0.55, 0.69) | 0.64 (0.56, 0.71) | 0.64 (0.58, 0.69) | 0.71 (0.6, 0.8) | 0.71 (0.62, 0.82) | 0.64 (0.58, 0.73) |
| **LR+ High** | 2.25 (1.0, 4.5) | 2.25 (1.2, 4.5) | 3.0 (1.0, 9.0) | 3.0 (1.0, 6.0) | 3.0 (1.0, 9.0) | 3.0 (1.0, 6.0) | 3.0 (1.0, 6.0) |
| **LR+ Inter** | 1.29 (0.0, 3.86) | 1.29 (0.32, 3.86) | 1.29 (0.43, 3.86) | 1.29 (0.43, 5.14) | 1.29 (0.43, 5.14) | 1.29 (0.43, 5.14) | 1.29 (0.43, 5.14) |
| **LR+ Low** | 3.3 (2.2, 4.4) | 3.3 (2.2, 4.4) | 3.3 (2.2, 4.4) | 3.3 (2.2, 4.4) | 3.3 (2.2, 4.4) | 3.3 (2.2, 4.4) | 3.3 (2.2, 4.4) |
| **LR- High** | 0.38 (0.0, 1.0) | 0.38 (0.0, 0.86) | 0.38 (0.0, 1.0) | 0.33 (0.0, 1.0) | 0.38 (0.0, 1.0) | 0.38 (0.0, 1.0) | 0.33 (0.0, 1.0) |
| **LR- Inter** | 0.92 (0.64, 1.5) | 0.92 (0.64, 1.29) | 0.86 (0.55, 1.29) | 0.8 (0.48, 1.29) | 0.86 (0.55, 1.29) | 0.86 (0.55, 1.29) | 0.86 (0.55, 1.29) |
| **LR- Low** | 0.49 (0.24, 0.55) | 0.49 (0.24, 0.55) | 0.49 (0.24, 0.55) | 0.49 (0.24, 0.55) | 0.49 (0.24, 0.55) | 0.49 (0.24, 0.55) | 0.49 (0.24, 0.55) |

Table S3. This table summarizes the performance results of an Random Forest (RF) model tested 10,000 times with varying numbers of features, ranging from 6 to 12 features. The metrics used for evaluation include Accuracy (Acc), Area Under the Curve (AUC) for high, intermediate, and low prediction categories, and Positive Likelihood Ratio (LR+ ) for high, intermediate, and low prediction categories. For each feature configuration, the table presents the metric value and the 95% confidence interval (CI). These values indicate the model's performance with statistical details, providing insight into the variability and reliability of the RF model's performance estimates under different testing conditions.

|  | **12 features** | **11 features** | **10 features** | **9 features** | **8 features** | **7 features** | **6 features** |
| --- | --- | --- | --- | --- | --- | --- | --- |
| **Acc** | 0.71 (0.62, 0.79) | 0.71 (0.62, 0.75) | 0.71 (0.62, 0.75) | 0.71 (0.62, 0.79) | 0.71 (0.62, 0.79) | 0.71 (0.62, 0.79) | 0.71 (0.62, 0.79) |
| **AUC High** | 0.81 (0.67, 0.92) | 0.81 (0.68, 0.92) | 0.81 (0.67, 0.92) | 0.81 (0.67, 0.94) | 0.81 (0.68, 0.94) | 0.83 (0.7, 0.94) | 0.83 (0.7, 0.94) |
| **AUC Intermediate** | 0.49 (0.4, 0.59) | 0.49 (0.4, 0.57) | 0.49 (0.4, 0.59) | 0.5 (0.4, 0.6) | 0.54 (0.44, 0.63) | 0.54 (0.43, 0.63) | 0.56 (0.45, 0.66) |
| **AUC Low** | 0.78 (0.7, 0.84) | 0.77 (0.69, 0.83) | 0.78 (0.7, 0.84) | 0.78 (0.69, 0.84) | 0.76 (0.68, 0.82) | 0.78 (0.69, 0.84) | 0.74 (0.64, 0.82) |
| **LR+ High** | 4.5 (2.0, 12.0) | 4.5 (2.0, 12.0) | 4.5 (2.0, 9.0) | 4.5 (2.0, 12.0) | 4.5 (2.0, 12.0) | 4.5 (2.0, 12.0) | 3.0 (1.5, 6.0) |
| **LR+ Inter** | 1.29 (0.64, 2.57) | 1.29 (0.43, 2.57) | 1.29 (0.43, 1.93) | 1.29 (0.43, 2.57) | 1.29 (0.43, 3.86) | 1.29 (0.43, 3.86) | 1.93 (0.64, 285715.95) |
| **LR+ Low** | 3.3 (2.2, 4.4) | 3.3 (2.2, 4.4) | 3.3 (2.2, 4.4) | 3.3 (2.2, 4.4) | 3.3 (2.2, 4.4) | 3.3 (2.2, 4.4) | 3.3 (2.2, 4.4) |
| **LR- High** | 0.3 (0.0, 0.67) | 0.3 (0.0, 0.67) | 0.3 (0.0, 0.67) | 0.3 (0.0, 0.67) | 0.3 (0.0, 0.67) | 0.3 (0.0, 0.67) | 0.33 (0.0, 0.75) |
| **LR- Inter** | 0.86 (0.64, 1.29) | 0.86 (0.64, 1.29) | 0.92 (0.64, 1.29) | 0.86 (0.55, 1.29) | 0.86 (0.55, 1.29) | 0.86 (0.55, 1.29) | 0.73 (0.48, 1.1) |
| **LR- Low** | 0.49 (0.24, 0.55) | 0.49 (0.24, 0.55) | 0.49 (0.24, 0.55) | 0.49 (0.24, 0.55) | 0.49 (0.24, 0.55) | 0.49 (0.24, 0.55) | 0.49 (0.24, 0.55) |

Table S4. This table summarizes the performance results of an Support Vector Machine (SVM) model tested 10,000 times with varying numbers of features, ranging from 6 to 12 features. The metrics used for evaluation include Accuracy (Acc), Area Under the Curve (AUC) for high, intermediate, and low prediction categories, and Positive Likelihood Ratio (LR+ ) for high, intermediate, and low prediction categories. For each feature configuration, the table presents the metric value and the 95% confidence interval (CI). These values indicate the model's performance with statistical details, providing insight into the variability and reliability of the SVM model's performance estimates under different testing conditions.

|  | **12 features** | **11 features** | **10 features** | **9 features** | **8 features** | **7 features** | **6 features** |
| --- | --- | --- | --- | --- | --- | --- | --- |
| **Acc** | 0.54 (0.54, 0.58) | 0.54 (0.5, 0.54) | 0.54 (0.54, 0.54) | 0.54 (0.54, 0.54) | 0.67 (0.62, 0.71) | 0.67 (0.62, 0.75) | 0.67 (0.58, 0.71) |
| **AUC High** | 0.62 (0.54, 0.71) | 0.62 (0.53, 0.69) | 0.62 (0.53, 0.71) | 0.62 (0.53, 0.71) | 0.68 (0.54, 0.81) | 0.75 (0.6, 0.88) | 0.71 (0.62, 0.81) |
| **AUC Intermediate** | 0.57 (0.49, 0.66) | 0.59 (0.49, 0.67) | 0.59 (0.48, 0.68) | 0.61 (0.48, 0.7) | 0.75 (0.67, 0.83) | 0.75 (0.65, 0.79) | 0.67 (0.6, 0.75) |
| **AUC Low** | 0.75 (0.63, 0.82) | 0.75 (0.62, 0.82) | 0.75 (0.64, 0.82) | 0.75 (0.64, 0.84) | 0.56 (0.42, 0.64) | 0.46 (0.38, 0.62) | 0.58 (0.4, 0.71) |
| **LR+ High** | 1.12 (1.0, 1.5) | 1.0 (0.9, 1.12) | 1.12 (1.0, 1.5) | 1.12 (1.0, 1.5) | 3.0 (1.5, 6.0) | 3.0 (1.0, 6.0) | 2.0 (1.0, 4.5) |
| **LR+ Inter** | 1.29 (0.43, 1.29) | 1.0 (0.0, 1.29) | 1.29 (0.43, 1.29) | 0.86 (0.43, 1.29) | 1.54 (1.29, 1.93) | 1.61 (1.29, 2.57) | 1.61 (0.96, 2.14) |
| **LR+ Low** | 2.2 (1.1, 2.2) | 1.1 (1.1, 2.2) | 2.2 (1.1, 2.2) | 1.1 (1.1, 2.2) | 1.1 (1.1, 2.2) | 2.2 (1.1, 2.2) | 2.2 (1.1, 2.2) |
| **LR- High** | 0.75 (0.5, 1.0) | 1.0 (0.75, 1.5) | 0.75 (0.5, 1.0) | 0.75 (0.5, 1.0) | 0.82 (0.55, 0.9) | 0.67 (0.3, 1.0) | 0.67 (0.3, 1.0) |
| **LR- Inter** | 0.96 (0.96, 1.29) | 1.0 (0.96, 1.12) | 0.96 (0.96, 1.29) | 1.07 (0.96, 1.29) | 0.32 (0.26, 0.51) | 0.32 (0.21, 0.77) | 0.64 (0.26, 1.03) |
| **LR- Low** | 0.88 (0.88, 0.98) | 0.98 (0.88, 0.98) | 0.88 (0.88, 0.98) | 0.98 (0.88, 0.98) | 0.98 (0.88, 0.98) | 0.88 (0.88, 0.98) | 0.88 (0.88, 0.98) |

Table S5. This table summarizes the performance results of an k-Nearest Neighbour (KNN) model tested 10,000 times with varying numbers of features, ranging from 6 to 12 features. The metrics used for evaluation include Accuracy (Acc), Area Under the Curve (AUC) for high, intermediate, and low prediction categories, and Positive Likelihood Ratio (LR+ ) for high, intermediate, and low prediction categories. For each feature configuration, the table presents the metric value and the 95% confidence interval (CI). These values indicate the model's performance with statistical details, providing insight into the variability and reliability of the KNN model's performance estimates under different testing conditions.

|  | **12 features** | **11 features** | **10 features** | **9 features** | **8 features** | **7 features** | **6 features** |
| --- | --- | --- | --- | --- | --- | --- | --- |
| **Acc** | 0.71 (0.62, 0.79) | 0.71 (0.62, 0.79) | 0.71 (0.62, 0.79) | 0.71 (0.62, 0.79) | 0.71 (0.62, 0.79) | 0.71 (0.62, 0.79) | 0.71 (0.62, 0.79) |
| **AUC High** | 0.81 (0.66, 0.96) | 0.82 (0.67, 0.96) | 0.81 (0.67, 0.96) | 0.82 (0.67, 0.96) | 0.81 (0.65, 0.96) | 0.81 (0.65, 0.96) | 0.83 (0.67, 0.98) |
| **AUC Intermediate** | 0.56 (0.45, 0.66) | 0.56 (0.45, 0.66) | 0.56 (0.45, 0.65) | 0.56 (0.45, 0.66) | 0.56 (0.44, 0.66) | 0.56 (0.44, 0.66) | 0.56 (0.44, 0.67) |
| **AUC Low** | 0.69 (0.51, 0.71) | 0.69 (0.51, 0.71) | 0.69 (0.51, 0.71) | 0.69 (0.51, 0.75) | 0.69 (0.51, 0.79) | 0.69 (0.51, 0.79) | 0.69 (0.51, 0.79) |
| **LR+ High** | 9.0 (3.0, 12.0) | 9.0 (3.0, 12.0) | 9.0 (3.0, 12.0) | 9.0 (3.0, 12.0) | 6.0 (2.25, 12.0) | 6.0 (3.0, 12.0) | 9.0 (3.0, 500000.82) |
| **LR+ Inter** | 1.29 (0.86, 2.57) | 1.29 (0.77, 2.57) | 1.29 (0.77, 2.57) | 1.29 (0.77, 2.57) | 1.29 (0.64, 2.57) | 1.29 (0.64, 2.57) | 1.29 (0.77, 2.57) |
| **LR+ Low** | 2.2 (1.1, 3.3) | 2.2 (1.1, 3.3) | 2.2 (1.1, 3.3) | 2.2 (1.1, 3.3) | 2.2 (1.1, 4.4) | 2.2 (1.1, 4.4) | 2.2 (1.1, 4.4) |
| **LR- High** | 0.27 (0.0, 0.6) | 0.27 (0.0, 0.6) | 0.27 (0.0, 0.6) | 0.27 (0.0, 0.6) | 0.27 (0.0, 0.67) | 0.27 (0.0, 0.6) | 0.27 (0.0, 0.6) |
| **LR- Inter** | 0.77 (0.55, 1.29) | 0.77 (0.55, 1.29) | 0.77 (0.55, 1.29) | 0.77 (0.55, 1.29) | 0.77 (0.55, 1.29) | 0.77 (0.55, 1.29) | 0.77 (0.51, 1.29) |
| **LR- Low** | 0.73 (0.49, 0.98) | 0.73 (0.49, 0.98) | 0.73 (0.49, 0.98) | 0.73 (0.49, 0.98) | 0.55 (0.24, 0.98) | 0.55 (0.24, 0.98) | 0.73 (0.24, 0.98) |

Table S6. This table summarizes the performance results of an Radial Basis Function (RBF) model tested 10,000 times with varying numbers of features, ranging from 6 to 12 features. The metrics used for evaluation include Accuracy (Acc), Area Under the Curve (AUC) for high, intermediate, and low prediction categories, and Positive Likelihood Ratio (LR+ ) for high, intermediate, and low prediction categories. For each feature configuration, the table presents the metric value and the 95% confidence interval (CI). These values indicate the model's performance with statistical details, providing insight into the variability and reliability of the RBF model's performance estimates under different testing conditions.

|  | **12 features** | **11 features** | **10 features** | **9 features** | **8 features** | **7 features** | **6 features** |
| --- | --- | --- | --- | --- | --- | --- | --- |
| **Acc** | 0.62 (0.58, 0.67) | 0.71 (0.62, 0.75) | 0.71 (0.62, 0.79) | 0.67 (0.58, 0.71) | 0.71 (0.62, 0.75) | 0.67 (0.62, 0.71) | 0.71 (0.62, 0.75) |
| **AUC High** | 0.69 (0.52, 0.73) | 0.56 (0.5, 0.69) | 0.75 (0.65, 0.83) | 0.81 (0.75, 0.85) | 0.67 (0.54, 0.75) | 0.73 (0.65, 0.77) | 0.92 (0.85, 0.94) |
| **AUC Intermediate** | 0.71 (0.63, 0.78) | 0.7 (0.63, 0.75) | 0.7 (0.62, 0.75) | 0.68 (0.6, 0.75) | 0.67 (0.59, 0.73) | 0.67 (0.6, 0.73) | 0.71 (0.67, 0.76) |
| **AUC Low** | 0.76 (0.71, 0.84) | 0.67 (0.6, 0.73) | 0.71 (0.65, 0.76) | 0.64 (0.55, 0.73) | 0.71 (0.65, 0.78) | 0.6 (0.51, 0.71) | 0.75 (0.69, 0.78) |
| **LR+ High** | 2.25 (1.0, 4.5) | 3.0 (0.0, 6.0) | 3.0 (1.0, 9.0) | 1.71 (1.33, 2.4) | 250000.92 (1.0, 250001.46) | 250000.93 (1.0, 250001.46) | 3.0 (2.0, 4.0) |
| **LR+ Inter** | 1.0 (1.0, 1.0) | 2.57 (1.29, 3.21) | 2.14 (1.29, 3.86) | 1.93 (0.64, 5.14) | 1.54 (1.1, 1.93) | 1.29 (1.29, 1.93) | inf (0.0, nan) |
| **LR+ Low** | 2.2 (1.47, 2.93) | 2.2 (1.32, 3.3) | 2.2 (1.1, 4.4) | 200001.0 (200000.59, 200001.39) | 2.2 (1.1, 3.3) | 1.1 (0.73, 2.2) | 2.93 (1.65, 6.6) |
| **LR- High** | 0.38 (0.3, 1.0) | 0.6 (0.5, 1.12) | 0.6 (0.27, 1.0) | 0.0 (0.0, 0.0) | 0.75 (0.75, 1.0) | 0.75 (0.75, 1.0) | 0.0 (0.0, 0.0) |
| **LR- Inter** | 1.0 (1.0, 1.0) | 0.55 (0.37, 0.86) | 0.43 (0.18, 0.86) | 0.8 (0.48, 1.1) | 0.32 (0.26, 0.64) | 0.43 (0.26, 0.43) | 0.86 (0.71, 1.12) |
| **LR- Low** | 0.0 (0.0, 0.44) | 0.55 (0.49, 0.73) | 0.73 (0.55, 0.98) | 0.8 (0.8, 0.8) | 0.73 (0.49, 0.98) | 0.98 (0.55, 1.1) | 0.27 (0.24, 0.63) |
